# Check the gap: Facemask performance and exhaled aerosol distributions around the wearer

**DOI:** 10.1371/journal.pone.0243885

**Published:** 2020-12-16

**Authors:** Emily L. Kolewe, Zachary Stillman, Ian R. Woodward, Catherine A. Fromen

**Affiliations:** Department of Chemical and Biomolecular Engineering, University of Delaware, Newark, Delaware, United States of America; VIT University, INDIA

## Abstract

Current facemask research focuses on material characterization and efficiency; however, facemasks are often not tested such that aerosol distributions are evaluated from the gaps in the sides, bottom, and nose areas. Poor evaluation methods could lead to misinformation on optimal facemasks use; a high-throughput, reproducible method which illuminates the issue of fit influencing aerosol transmission is needed. To this end, we have created an *in vitro* model to quantify particle transmission by mimicking exhalation aerosols in a 3D printed face-nose-mouth replica via a nebulizer and quantifying particle counts using a hand-held particle counter. A sewn, sewn with pipe cleaner nose piece, and sewn with a coffee filter facemask were used to evaluate current common homemade sewn facemask designs, benchmarked against industry standard surgical, N95 respirator tightly fit, and N95 respirator loosely fit facemasks. All facemasks have significantly reduced particle counts in front of the facemask, but the side and top of the facemask showed increases in particle counts over the no facemask condition at that same position, suggesting that some proportion of aerosols are being redirected to these gaps. An altered size distribution of aerosols that escape at the vulnerable positions was observed; escaped particles have larger count median diameters, with a decreased ratio of smaller to larger particles, possibly due to hygroscopic growth or aggregation. Of the homemade sewn facemasks, the facemask with a coffee filter insert performed the best at reducing escaped aerosols, with increased efficiency also observed for sewn masks with a pipe cleaner nose piece. Importantly, there were minimal differences between facemasks at increasing distances, which supports that social distance is a critical element in reducing aerosol transmission. This work brings to light the importance of quantifying particle count in positions other than directly in front of the facemask and identifies areas of research to be explored.

## Introduction

The Centers for Disease Control and Prevention (CDC), World Health Organization (WHO), and other health organizations around the world have encouraged or required the use of facemasks to slow the spread of respiratory diseases [1, 2]. These recommendations are built on growing epidemiological population studies demonstrating that communities with high facemask compliance have been effective in reducing case numbers and disease reproduction number (R) of many viral respiratory diseases [3, 4]. COVID-19, influenza, and other respiratory diseases are spread through respiratory droplets (defined by the WHO as exhaled particles greater than 5 μm in size) and aerosols (also known as droplet nuclei, defined by the WHO as exhaled particles smaller than 5 μm in size) [5–7]. This definition is not universal, however, with the aerosol community defining the entirety of suspended solid and liquid particles as aerosols, which would encompass the WHO’s definition of both respiratory droplets and aerosols and also include larger particles as well [8, 9]. In the context of preventing viral infection, facemasks may serve a dual purpose in limiting airborne viral transmission through reduction of aerosol exhalation and also of aerosol inhalation (facemask definitions expanded in Supplemental Information). Some masks, such as the N95 respirator, protect the wearer from inhaling aerosols, while other masks are primarily intended to prevent the exhalation of aerosols from the wearer [8, 10, 11] and can include an increasing number of homemade or commercial sewn facemasks, whose efficacy for reduction of exhaled particles remains under investigation [12].

Work in the 20th century honed facemasks to the structured and high filtration efficiency masks we know today, such as the N95 respiratory and surgical facemasks [13, 14]. These initial studies crucially set a framework for evaluating critical facemask properties, such as conformation to face, material efficiency, and aerosol size variations [13, 14]. However, the earliest mentions of homemade facemasks for the purposes of protecting large populations from pandemics were not published on until the early 1980’s [15, 16]. In recent studies evaluating homemade facemasks, one of the most investigated topics with regard to facemasks is the choice of material: cotton, polyester, lint-free materials, and others, in addition to homemade filters from coffee filters, nylon, and more [4, 8, 17, 18]. Most of the provided guidelines in designing a facemask to reduce transmission of exhaled aerosols are based the material’s filtration efficiency [4, 8, 17, 18]. Materials made from fibers have a few main mechanisms of filtering particles: diffusion (small, slow particles eventually contacting filter fibers), interception (particles following an air stream directly in the path of a filter fiber), impaction (large particles exiting an air stream and contacting a filter fiber), and electrostatic attraction (polar forces pull particles towards filter fibers) [19–21]. Given the turbulent flow profile and facemask resistance to airflow, air may not entirely penetrate through the facemask and instead may be redirected around the facemask to gaps, commonly at the nose, cheeks, and chin, where facemask material tends to crease and allow air to escape [22]. The presence or lack of gaps is referred to as facemask ‘fit’ and can be measured either quantitatively (via destructive particle counting inside and outside of a facemask at a reference single location) or from a qualitative measure of a wearer being able to smell or taste ambient particles [23, 24]. Descriptions of fit have also been related to particle size [19, 25], intended facemask use [23], and gap size in material [8]. To date, most evaluations do not simultaneously provide insight into the location of leaks/gaps in the fit, quantitation of those leaks/gaps, aerosol distributions at discrete locations, inclusion of human face shape, and high-throughput sampling [4, 8, 17, 26–28]. Computational simulations have shown that exhaled aerosols may travel several feet even during facemask usage, and it is clear that air flow is redirected around the facemask; yet these “gaps” are not easily included in facemask efficacy calculations [10, 27, 29]. As a result, there are limitations to our current understanding of facemask evaluation, design, and usage, especially in regard to limiting exhaled particulates.

In this work, we aim to implement a high-throughput method of quantifying aerosol distributions following flow through facemask gaps to gain a more holistic view of exhaled aerosol transmission during the use of facemasks. To this end, we have created an *in vitro* model using a 3D printed face replica with an attached nebulizer capable of mimicking aerosol exhalation to evaluate the aerosol transmission at different locations around the face following use of various sewn and industry-standard facemasks. We confirm that all facemasks significantly reduce particle counts directly in front of the facemask, but the side and top of the facemask show increases in particle counts over the No Mask scenario, suggesting that particles are not completely filtered but rather are redirected to these gaps. Interestingly, we observe a shift in exhaled particle size distribution, implying the increased residence time provided by the facemask can promote aerosol evolution and opportunity for hygroscopic growth. Importantly, there were minimal differences between facemasks at increasing distances away from the face. This work brings to light the importance of quantifying particle count in positions other than directly in front of the facemask and identifies areas of research to be explored. By focusing on a range of common facemask materials at varied pressure drop and collection efficiencies, our approach can provide evaluation of fit in addition to material filter efficiency and, in the long-term, support evaluation of inherent variability from human subjects to evaluate fit with higher experimental throughput.

## Materials and methods

### Facemasks

**Fig 1:**
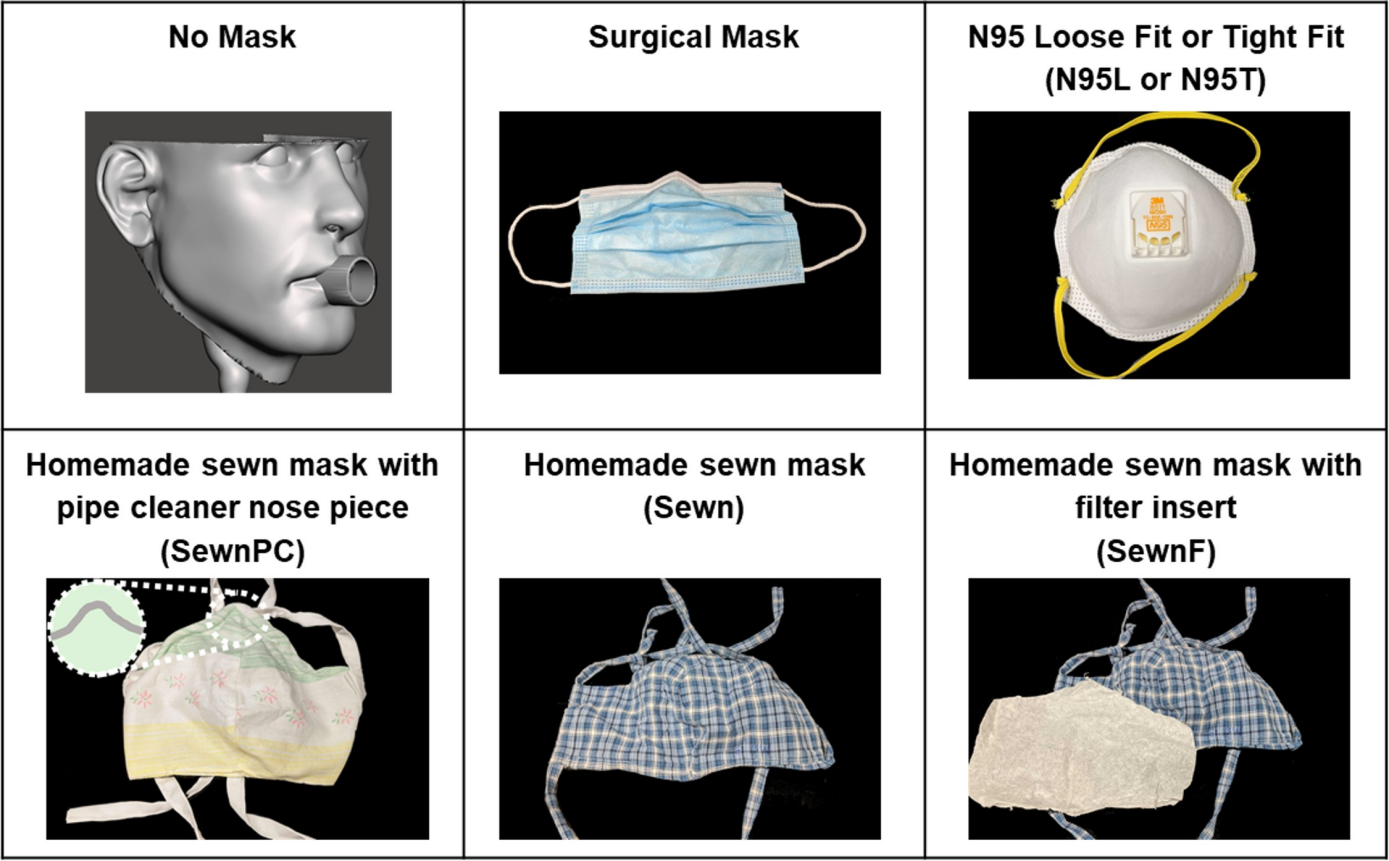
Images of facemasks used in this study. Each facemask is listed along with its in-text abbreviation (figure label) and an image of the facemask type.

The facemasks evaluated in this study include both commercially available facemasks and homemade sewn alternatives to represent likely options for the general population, as shown in **Fig 1**. Commercial facemasks include a surgical facemask with nose clip (Surgical mask) and an N95 respirator tested with a loose fit (N95L) and an artificially applied tighter fit (N95T). Homemade sewn facemasks include a two-layer, hand-sewn facemask (Sewn), two-layer, hand-sewn facemask with an interstitial coffee filter (SewnF), two-layer, hand-sewn facemask with a pipe cleaner nose clip (SewnPC). All hand-sewn facemasks were made from common household cotton fabric. Facemasks are shown in **Fig 1**.

### 3D printing

All custom parts for the face replica and pressure experiments were printed using a Carbon M1 3D printer (Carbon Inc., Redwood City, CA) with 100 μm slicing in the vertical print direction and utilized the Dynamic Light Synthesis (DLS) setting. The proprietary resin materials used for each part include: elastomeric polyurethane (EPU 40) for flexible adapters and plugs, urethane methacrylate (UMA 90) for anatomical replicas, and prototyping resin (PR 25) for pressure drop test nozzles. Parts were prepared according to the manufacturer’s recommendations. For parts printed in PR 25 or UMA 90, parts were cleaned with isopropyl alcohol (IPA) before being exposed to ultraviolet light to cure remaining resin for 30 second intervals until all areas were exposed and parts were no longer tacky. For parts printed in EPU, parts were cleaned briefly with IPA for 1 minute before being baked to cure remaining resin for 8 hours.

### Face and airway replica

An idealized face 3D model was used and analyzed, described in **Supplemental Information Methods** and shown in **Fig A in S1 File**. This was aligned and connected to an upper airway model including the trachea, oral airway, and nasal airway. The idealized upper airway of Feng *et al*. [30] was modified by incorporating an idealized nasal passage and sampling ports designed to accommodate the particle counter at the back of the mouth and the trachea. Upper airways were 3D printed in a single continuous part, and the face was printed in three parts due to print volume constraints. All parts were assembled and joined using a liquid gasket sealant. To control airflow and conserve particles within the replica during testing, plugs were designed for the sampling ports, mouth, and nostrils. All parts were fitted and combined using Solidworks 2019 (Dassault Systems, Vélizy-Villacoublay, France) and Meshmixer (Autodesk, San Rafael, CA). For the purposes of this study, only the mouth outlet (OD = 20 mm) was left open during measurements.

### Particle generation and counting

**Fig 2:**
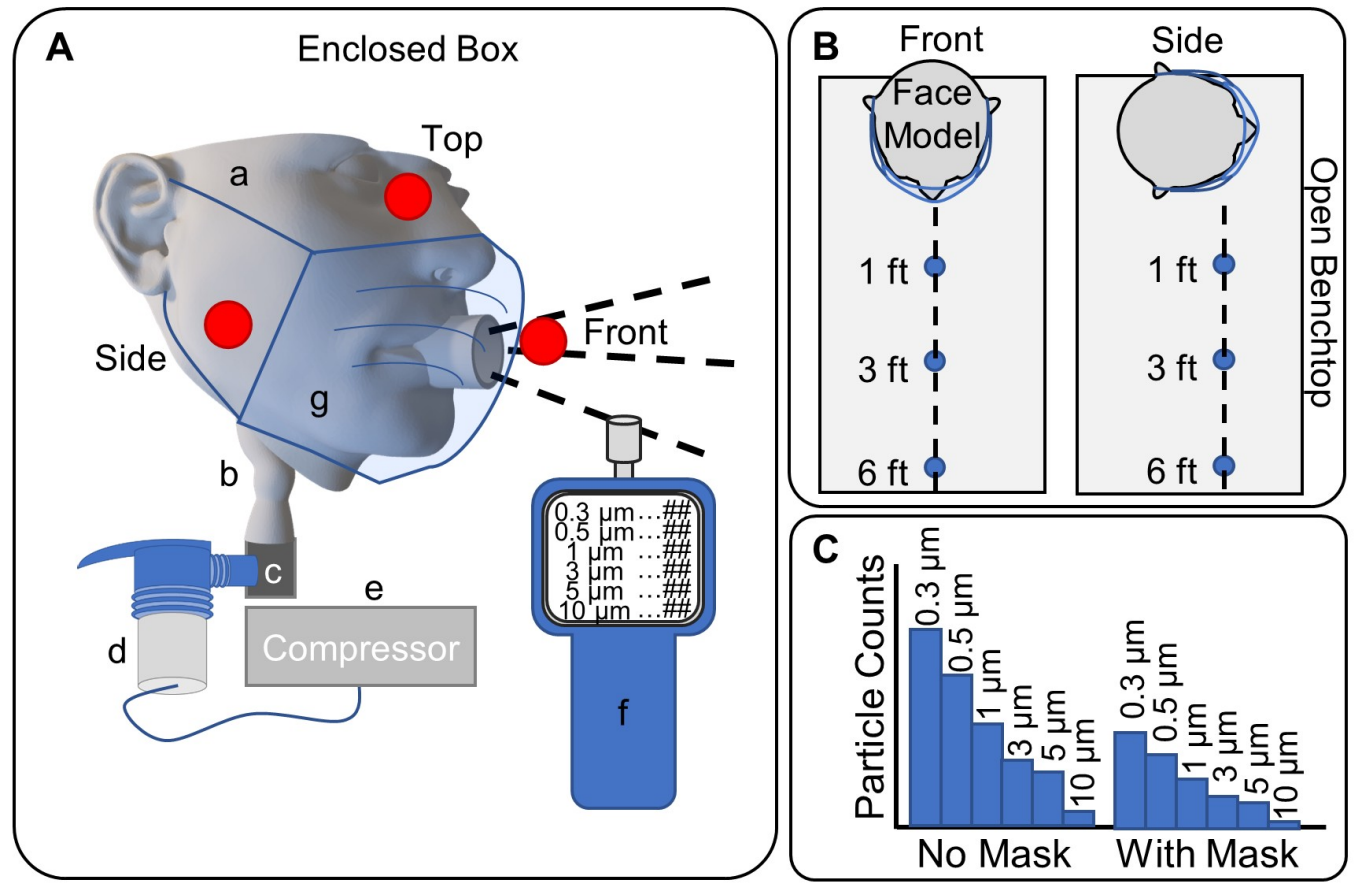
Schematic of experimental set-up. **A.** Schematic of the enclosed box experimental setup showing the (a) face model, (b) idealized airway replica, (c) throat adapter, (d) PARI LC Sprint Nebulizer, (e) Vios compressor, (f) particle counter, and (g) facemask. Additionally, the locations where particle counts were measured are represented by red dots; specifically, the Front, Side, and Top. **B.** Schematic of the open bench experimental setup, showing the face model with a facemask, connected to the same nebulizer (not shown), and the distances at which particles were measured with the particle counter: one foot, three feet, and six feet (0.3 m, 0.9 m, and 1.8 m), in both the Front and Side positions. **C.** Representative example of the data collected at each sample, with particle size bins for 0.3, 0.5, 1, 3, 5, and 10 μm particles grouped together in increasing order such that each grouping represents a facemask and position, such as No Mask and With Mask for one foot (0.3 m) from the model.

Model exhaled aerosolized particles were generated using a PARI Vios Aerosol Delivery System and LC Sprint nebulizer (PARI, Starnberg, Germany) fitted to the trachea replica with a flexible 3D printed adapter (**Fig 2**). The nebulizer compressor pumps 50 psi (345 kPa) air continuously over the duration of an experiment, which travels through the nebulizer (aerosolizing particles) to the airway model to mimic exhalation [31]. The device is powered on just prior to sampling and powered off after sampling is complete, before removing the facemask and preparing for the next treatment. Water with no added solid contents was selected as the particle medium to simulate natural respiratory emission. The nebulizer was run continuously with water added to maintain a visible level, ensuring a consistent size distribution and concentration of generated aerosols. The flow rate exiting the mouth was measured at 17 L/min (see **Supplemental Methods** and **Fig C in S1 File**). Particle counting was performed utilizing a TSI AeroTrak Handheld Particle Counter Model 9306 (TSI Inc., Shoreview, MN) over a 10 second period with three independent replicates collected by the counter for each covering (or lack thereof), position, and environmental condition. This device can detect single counts at 50% efficiency for 0.3 μm particles and 100% efficiency above 0.45 μm, up to 2.1x10^8^ particles/m^3^. The particles counted were in bins of 0.3–0.5 μm, 0.5–1 μm, 1–3 μm, 3–5 μm, 5–10 μm, and larger than 10 μm. The counter sampled 0.5 L of air over the 10 s period and was placed at various positions relative to the face/mouth opening. Two types of environmental conditions were performed to measure particle emission 1) within an enclosed plexiglass box and 2) within an open room environment on a benchtop, as described in the following. Aerosols were generated and quantified in a climate-controlled environment maintained at an average of 21°C and 40–50% relative humidity. No modifications were made to the nebulizer or 3D printed apparatus that would influence the temperature of the aerosol or ambient air. Before and after experiments, which occurred over the course of ~3 hours, background levels of aerosols were taken in front of the 3D printed replica, in triplicate, under representative experimental conditions. Within each set of experiments, measurements were taken at equivalent positions from the face for the following coverings: No Mask, Sewn, SewnF, SewnPC, N95L, N95T, and Surgical facemasks.

### Experimental setup: Enclosed box

The first set of particle-counting measurements was made by placing the 3D printed face model inside of a plexiglass box (17 in x 17 in x 17 in; 0.4 m x 0.4 m x 0.4 m) with aerosolized particles being fed through the trachea of the face model. An additional 3D printed feedthrough adapter held the face replica in place while sealing the plexiglass box. During measurement, the particle counter was placed manually at the sampling locations within the box at locations indicated in **Fig 2A**. The coordinates within the box of the three positions are (8.5 in, 5.5 in, 2 in; 2.2 x 10^−1^ m, 1.3 x 10^−1^ m, 5.1 x 10^−2^ m) for the Front position, (1.5 in, 5 in, 2 in; 3.8 x 10^−2^ m, 1.3 x 10^−1^ m, 5.1 x 10^−2^ m) for the Side position, and (8.5 in, 6 in, 7.5 in; 2.2 x 10^−1^ m, 1.5 x 10^−1^ m, 1.9 x 10^−1^ m) for the Top position, all of which are coordinates relative to the front left corner on the bottom of the box. For reference, the mouth opening was positioned at (8.5 in, 9 in, 2 in; 2.2 x 10^−1^ m, 2.3 x 10^−1^ m, 5.1 x 10^−2^ m). Positions were selected based on locations of particle streams from some masks with others at background levels or were approximately equivalent to those selected.

### Experimental setup: Open bench

The second set of particle-counting measurements was made in an open environment by securing the 3D printed face model to a benchtop ring stand. The face replica was positioned at one end of a lab bench in the center of an open 440 ft^2^ (41 m^2^) laboratory space, and the particle counter was placed at various points on the bench including Front, Side, and vulnerability positions (localized particle streams that were visually observed and experimentally confirmed). To limit the effects of airflow and room obstacles on measurement readings, the particle counter remained stationary on the bench while the replica was rotated to the Front or Side position (see **Fig 2B**). In addition to these position changes, measurements were also taken at distances of three-feet (0.9 m) and six-feet (1.8 m) away from the face model to mimic social distancing environments. As in the first setup, the particles were generated by a PARI nebulizer system and fed through the trachea replica before exiting the mouth.

### Experimental setup: Pressure drop

For each of the facemasks, the pressure drop across the material was determined using a mass flow meter Model 4043 (TSI Inc., Shoreview, MN), flow controller Model TPK 2000 (Copley, Colwick, Nottingham, UK), and vacuum pump Model SCP5 (Copley, Colwick, Nottingham, UK). A 3D printed assembly was designed to accommodate a single layer of facemask material and hold it taut during measurement, where the assembly was fitted to the flow meter. An example of the setup is shown in **Fig B in S1 File**. For each measurement, vacuum was applied to generate a flow rate of 30 standard liters per minute (SLPM, set before attaching the sample) for 20 seconds (in triplicate).

### Statistical analysis

Data were analyzed using and R v3.6.3 (R Core Team, Vienna, Austria). Univariate and multivariate analysis of variance was performed using the [stats] package [32], and post-hoc multiple comparisons tests with 95% confidence intervals were performed with Tukey’s adjustment using the [emmeans] package [33]. [tidyverse] [34] packages were used in the preparation and presentation of the data. Background-corrected aerosol levels were calculated by taking the difference of measured aerosol levels and the average day-specific background aerosol levels for each size bin; for open bench measurements, the start-of-day levels were used for subtraction. All analyses were performed on background-corrected aerosol levels without transformation. Count Median Diameters (CMDs) for aerosol distributions were calculated using the cumulative size distribution of averaged replicates and represent the fraction of particles smaller than reported value [35]. Details of the statistical results are reported in the Supplementary Information.

## Results

**Fig 3:**
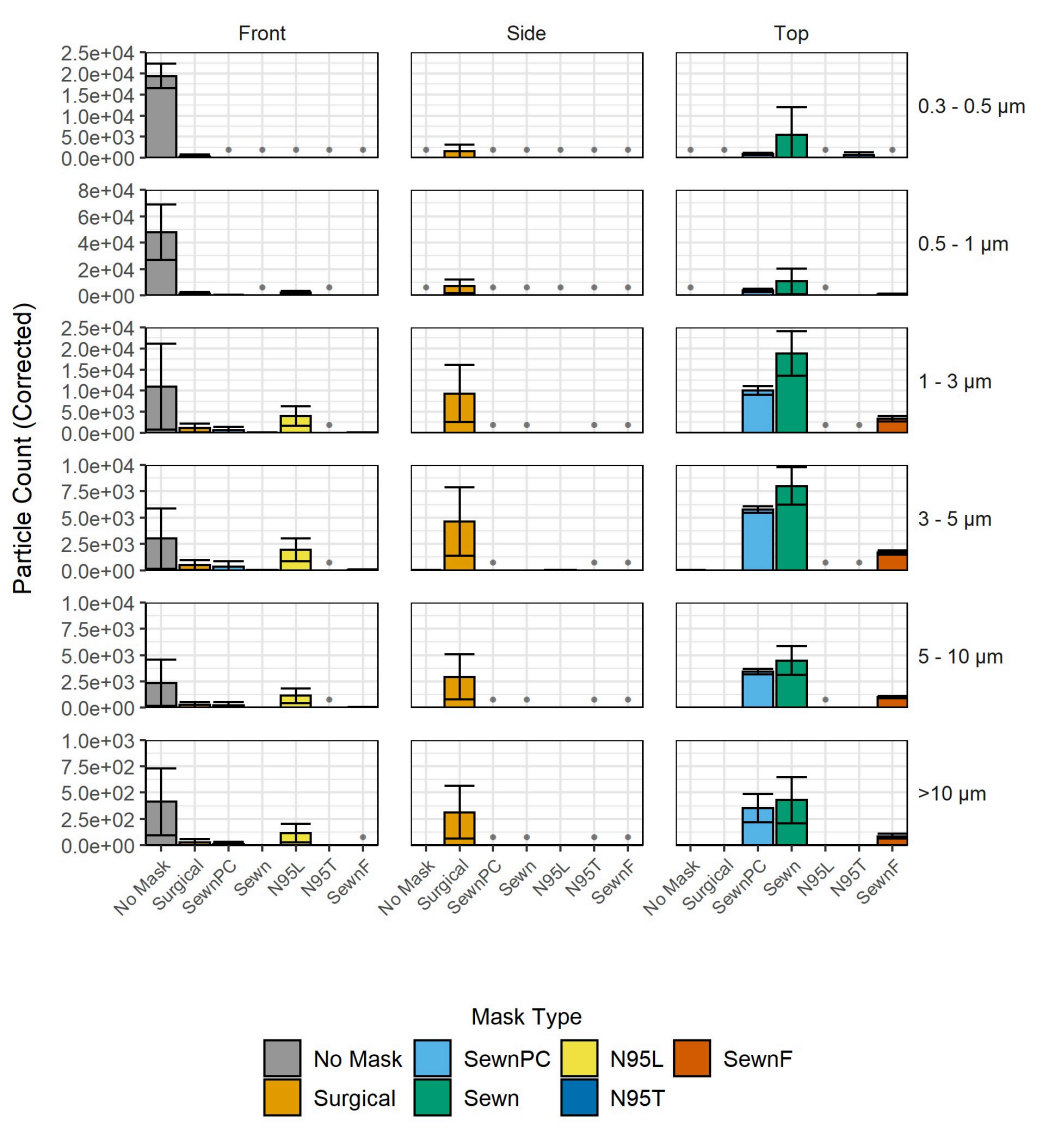
Background-corrected particle counts comparing facemask treatments within the enclosed box. Measurements shown in each row for the six particle size bins are shaded and grouped by facemask type, including No Mask, Sewn, SewnF, SewnPC, Surgical, N95L and N95T. Axes are scaled to each respective range, and gray bullet points at the baseline indicate negative corrected counts. Error bars indicate the sample standard deviation for particle counts in each of the respective particle size bins from three independent measurements. A 2-factor MANOVA and subsequent 2-factor ANOVAs were performed in addition to Tukey's Honest Significant Difference tests; p-values, relevant parameters, and analysis are shown as interactive maps and graphs included in **S2 File**.

Background-corrected particle levels for the enclosed box experiments are shown in **Fig 3**. In cases where the background-corrected particle count was negative (below background), these measurements are denoted with a single gray dot at the baseline. All counts in each particle size bin from the No Mask, Front position registered at least one order of magnitude above background, and these values were taken as the reference point for maximum aerosol exposure. All facemask conditions decreased the aerosol transmission level, the total number of aerosols escaping the facemask, relative to maximum in the 0.3–0.5 μm and 0.5–1 μm size bins. Compared to the Front position, some facemasks showed increased numbers of particle transmission at the Top position (Sewn, SewnPC, SewnF) or Side position (Surgical). Some facemasks also demonstrated a redistribution of the relative number of particles in each size bin, shifting towards particle sizes >1 μm. This shift is reflected in the calculated CMD at each position for the respective masks, shown in **Table A in S1 File**. The No Mask condition showed a high aerosol count in the 0.5–1 μm size bin at the Front position and a CMD of 0.74 μm, while all other facemasks showed decreased aerosol levels in the 0.3–0.5 μm and 0.5–1 μm size bins with a corresponding increase in CMD, with the exception of the N95T respirator. In all locations with detectable particle concentrations (Front, Side, and Top), all facemasks demonstrated an increase in CMD relative to the No Mask, Front position with the exception of the N95T mask, further supporting a shift in size particle distribution. Sewn cloth facemasks exhibited higher aerosol levels at the Top position, with the SewnPC mask having particle counts comparable to the Sewn mask, both of which had higher particle counts than the SewnF mask. Each of the Sewn, SewnPC, and SewnF masks exhibited the highest aerosol counts in the 1–3 μm size bin at the Top position with CMDs between 1.84 and 2.54 μm at this position. In comparison, the Surgical mask showed the highest transmission in the 1–3 μm size bin at the Side position with a CMD of 1.85 μm at this position. Finally, N95 performance differed greatly between the two fit conditions. The N95L respirator showed highest aerosol counts in the 1–3 μm size bin at the Front position but as much as 65% average transmission in the 3–5 μm size bin at the Front position relative to the No Mask condition. However, the N95T achieved <5% average transmission for all particle sizes at the Front position relative to the No Mask condition, leading to comparatively low particle sample sizes used to calculate CMD and possibly contributing to the low CMD value. Both N95 conditions limited aerosol transmission at the Top and Side positions.

**Fig 4:**
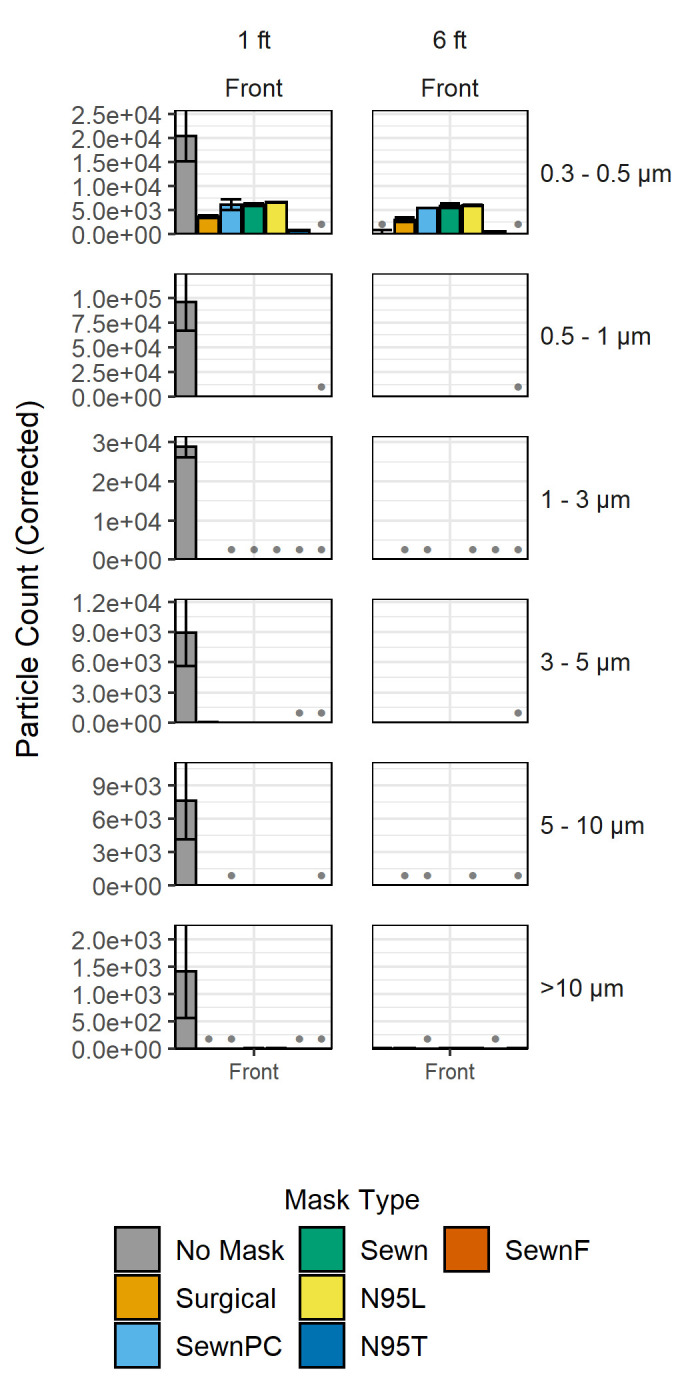
Background-corrected particle counts comparing facemask treatments in the open bench environment. Each plot shows background-corrected particle counts for each of the facemasks, which are ordered from least to greatest pressure drop, at distances of one foot, three feet, and six feet (0.3 m, 0.9 m, and 1.8 m) away from the face model. The Front position is shown for each distance. Binning by particle diameter for each facemask is grouped together by row, in order of size from 0.3–10 μm, as described in **Fig 2**. Axes are scaled to each respective range, and gray bullet points at the baseline indicate negative corrected counts. Error bars indicate standard deviation for particle counts in each of the respective particle size bins from three independent measurements. A 3-factor MANOVA and subsequent 3-factor ANOVAs were performed in addition to Tukey's Honest Significant Difference tests p-values, relevant parameters, and analysis are shown as interactive maps, and contour plots and graphs of data from one foot, three feet, and six feet (0.3 m, 0.9 m, and 1.8 m) in the Front and Side positions are included in **S3 File**.

Following evaluation of aerosol transmission in the more controlled enclosed box environment, similar evaluations were performed to determine particle transmission in an open bench environment at one-foot, three-foot, and six-foot distances (0.3 m, 0.9 m, and 1.8 m). This served to more accurately capture the dynamics that would be encountered in an environment such as a workspace, school, or retail location, but is less controlled than the enclosed box environment. Particle counts were first measured on a bench one foot (0.3 m) away from the face model. When measured with No Mask, particle counts were 48-fold above background as can be seen in the first panel of **Fig 4**. There were also counts above background measured one foot (0.3 m) away from the model with the SewnF mask, 15-fold above background. All other counts at the one-foot (0.3 m) mark were very slightly above or below background at the Front position (in the range of 3-5-fold higher). The exception to this was the N95T respirator, which reduced total particle counts by 70% and particle counts reduced below background levels. Three feet (0.9 m) away from the model, all facemasks showed levels slightly above or below background, again with the exception of the N95T respirator that had particle counts reduced by 72% below background (see **Fig D in S1 File**). All other facemasks showed particle counts approximately at background levels at the three-foot (0.9 m) mark with counts less than 5-fold above background. Similarly, at six feet (1.8 m) away from the model, all facemasks showed levels slightly above or below background with the exception of the N95T respirator that had particle counts reduced 65.88% below background. This information is also represented visually in surface plots (see **S4 File**).

**Fig 5:**
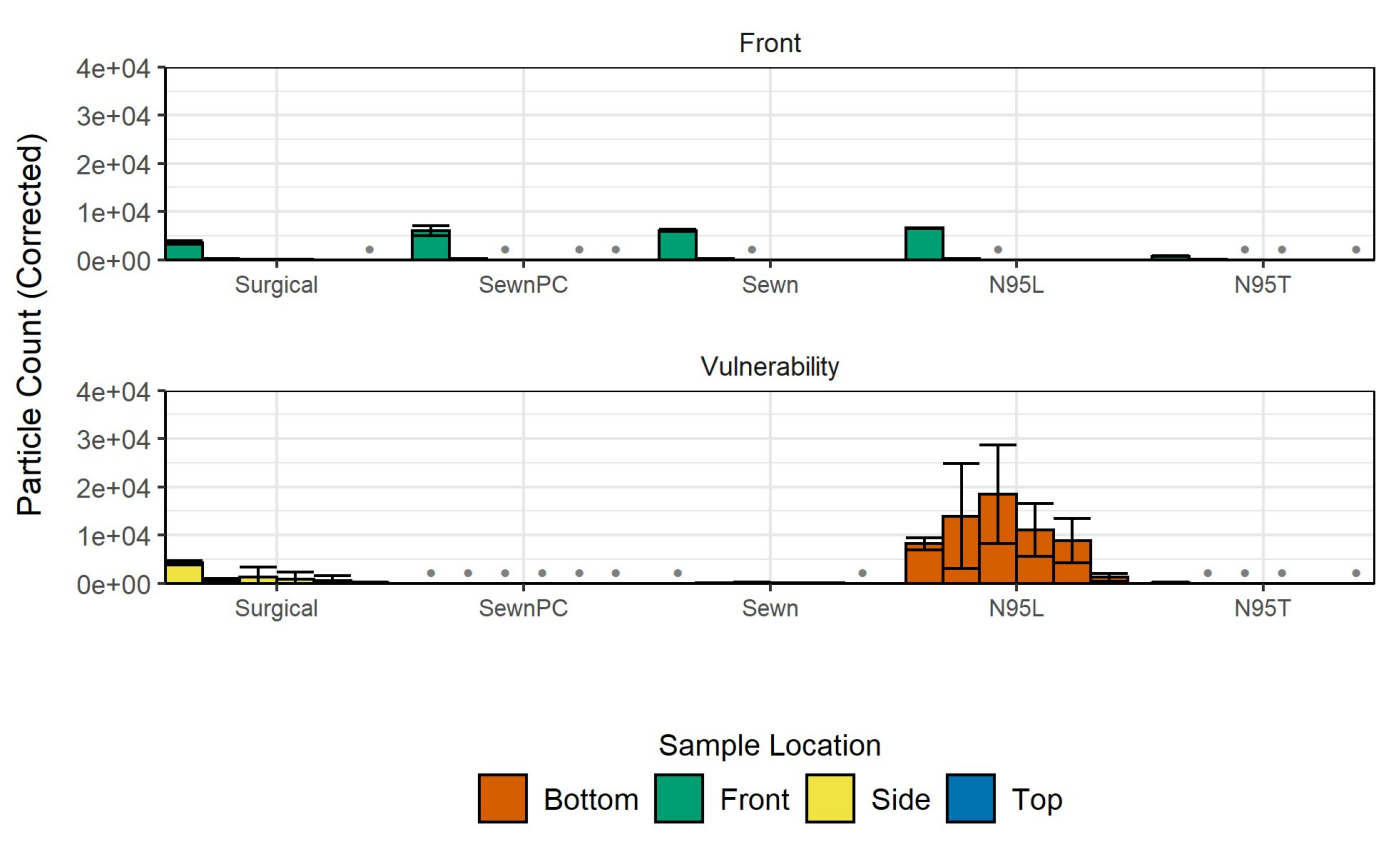
Background-corrected particle counts comparing Front and vulnerable positions for each facemask at a one-foot (0.3 m) distance in the open bench environment. Measurements shown are grouped by particle size bin and colored by measurement position. Row 1 includes particle counts for the Front position of each facemask. Row 2 includes vulnerable positions deemed significant during experimental observation. Vulnerable positions include: Side (Surgical mask), Bottom (N95L), and Top (sewn masks, N95T). Gray bullet points at the baseline indicate negative background-corrected counts. Error bars indicate the sample standard deviation for particle counts in each of the respective particle size bins from three independent measurements. For statistical analyses, refer to the open bench analysis described in **Fig 4**.

**Fig 6:**
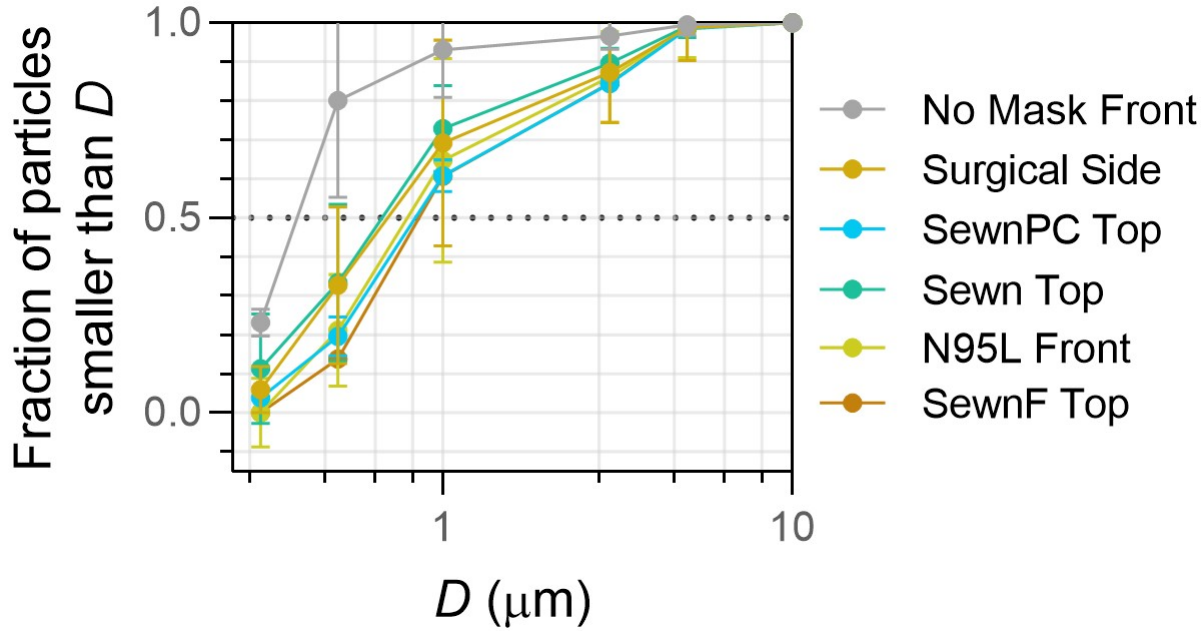
Background-corrected cumulative size distributions comparing distributions at vulnerable positions for each facemask in the enclosed box environment. Vulnerable positions include: Side (Surgical mask), Front (No Mask, N95L), and Top (sewn masks, N95T). Diameter (D) obtained from upper bin size and reported in μm. Error bars represent standard deviation for particle counts in each of the respective particle size bins from three independent measurements.

In observations from the enclosed box experiments and the open bench distance experiments, we noted that each facemask type presented unique gaps on the face model that allowed for particle escape at locations we termed as “vulnerabilities” (see **S1–S3 Videos**). Accordingly, comparisons of open bench particle counts were made for each facemask at their vulnerable positions determined by the position with largest particle counts aside from the Front position. Vulnerable positions were found to be the Top positions for all of the sewn masks and the N95T respirator, the Bottom position of the N95L respirator, and both the Side and Top positions for the Surgical mask. Non-vulnerable positions were not measured, as preliminary counts indicated that only vulnerable positions had counts above background. When evaluated at the vulnerable position, particle counts for the N95L and Surgical masks were elevated across almost all particle sizes relative to the Front position for the facemasks (**Fig 5**). This was not the case for the SewnPC and Sewn masks, which had lower counts at their vulnerable position relative to the Front position. The N95T and SewnF masks had approximately equivalent counts at both the Front and Vulnerability positions, indicating the effectiveness of the masks. The extent to which the facemasks allow for the transmission of particles relative to the No Mask case varied greatly from mask to mask, with the N95L allowing the greatest transmission of particles at its vulnerable location while the N95T, SewnPC, Sewn, and SewnF allowed relatively minimal transmission, even at their vulnerable position. The Surgical mask had intermediate levels of transmission at its vulnerable positions relative to the two previously mentioned groups. From the enclosed box experiments, vulnerable positions were found to have altered particle distributions (**Fig 6**) as compared to the No Mask size distribution, and also have elevated CMDs (**Table 1**).

**Table 1:** Count Median Diameters (CMD) of particles collected at vulnerable locations between facemask treatments in the enclosed box environment.

	Location	CMD
No Mask	Front	0.74
Surgical	Side	1.95
Sewn	Top	1.84
SewnF	Top	2.54
SewnPC	Top	2.48
N95L	Front	2.32
N95T	Top	<0.5

CMD in μm shown for each facemask type, including No Mask, Sewn, SewnF, SewnPC, Surgical mask, N95L and N95T at their respective vulnerability positions in the enclosed box environment.

**Fig 7:**
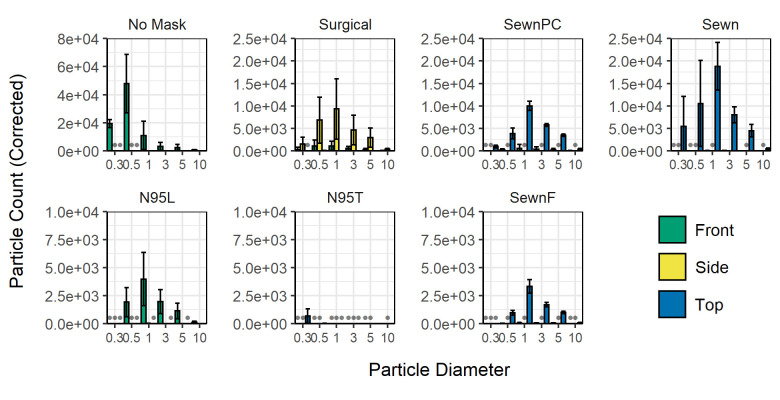
Background-corrected particle counts comparing facemask treatments within the enclosed box. Measurements shown for the six particle size bins are shaded and grouped by the measurement position (Front—Green, Left; Side—Yellow, Middle; Top—Blue, Right). Row 1 includes No Mask, Sewn, SewnF, SewnPC. Row 2 includes more advanced and commercially available facemask treatments (Surgical, N95L, and N95T masks). Gray bullet points at the baseline indicate negative corrected counts. Error bars indicate the sample standard deviation for particle counts in each of the respective particle size bins from three independent measurements. A 2-factor MANOVA and subsequent 2-factor ANOVAs were performed in addition to Tukey's Honest Significant Difference tests; p-values, relevant parameters, and analysis are shown as interactive maps and graphs included in **S2 File**.

Another property of interest that may affect the effectiveness of the facemasks is the pressure drop across each of the facemask materials, which was hypothesized to influence both breathability and filtration efficiency. Particle counts in the enclosed box environment are shown in **Fig 7** with the facemasks shown in order of increasing material pressure drop, as described in **Table 2**. The pressure drop across the facemask materials all had standard deviations less than 0.06 kPa and were statistically significantly different from each other. The material with the highest pressure drop was the SewnF mask and the material with the lowest was the Surgical mask. The paper coffee filter was measured as a comparison to these facemasks and had a pressure drop of 3.07 kPa, which contributes significantly to SewnF having the greatest pressure drop. The position of particles exiting differed between facemasks and did not trend with pressure drop; only the N95L respirator showed most significant particle counts in the Front position, only the Surgical mask showed most significant particle counts in the Side position, and all other facemasks showed most significant particle counts in the Top position. The facemask with the lowest particle counts in any position was the N95T respirator. The Sewn mask had greater particle counts than the SewnPC and the Surgical mask, which are materials with similar pressure drops and shape design. The N95L respirator had higher particle counts than the N95T respirator. The material with the highest pressure drop, the SewnF mask, had higher particle counts than the N95T respirator. While there were particle counts significantly above background for all masks, the counts were lower than not having a mask in all cases.

**Table 2:** Pressure drop across facemask materials.

Facemask	Surgical	SewnPC	Sewn	N95	SewnF
Pressure Drop (kPa)	0.5	0.63	1.2	1.4	3.07

The facemask measured and the respective pressure drop in kPa across the material. Measurements were taken at 17 L/min, n = 3, statistical analysis is reported in **Table B in S1 File**.

In addition to particle count measurements, the counts and particle diameters were translated to volumes of particles assuming spherical particles, as is described in the **Supplemental Methods** Aggregation Analysis in **S1 File**. Volumes of each particle size bin were summed to determine the total volume of particles measured for a given facemask and position, and subsequently divided by the total volume to report the volumetric fraction of each particle size for a given facemask and position. These relative volumetric fractions were normalized to the No Mask, Front position in the enclosed box environment, which effectively describes the distribution of particles based on volume relative to the No Mask, Front box environment condition. It can be shown that the average of the facemask-normalized relative volume fractions has a lower volume of small particle sizes, with volumes of 15% below background for 0.3 μm, and 91% and 19% below No Mask, Front box environment conditions for 0.5 and 1 μm particles, respectively. Mid-size particles had an increase in volumes, with 59% and 24% more 3 and 5 μm particles, respectively. The volume of 10 μm particles showed a decrease as well, with 26% fewer particles.

## Discussion

Historical studies have evaluated masks by primarily considering mask materials [36] and protection offered to the wearer during inhalation [37]. Furthermore, the development of respirator fit testing offered reliable, standardized assays and indication of common points of failure by improper fit [38]. Recent investigations into the behavior of exhaled aerosols have identified that, under the right conditions, they may migrate farther than the 6 ft (1.8 m) physical distancing guidelines [39]. However, through use of an N95 respirator, surgical mask, or other facemask,–even those which are not evaluated in a fit test scenario–aerosol transmission can be limited to distances under 6 ft (1.8 m) [40, 41]. In general, recent studies find that N95 respirators offer the best performance, followed by surgical masks and alternatives. With inconsistent fit characteristics between facemask types, physiological conditions, and wearer behavior, facemask performance can suffer, leading to a moderate amount of exhaled aerosols entering the ambient air [40–43].

We evaluated 7 facemask treatments (No Mask, Sewn, SewnPC, SewnF, Surgical, N95L, N95T) in enclosed box and open bench environments. To complement previous findings in the field, the focus of this study has been facemask types, rather than mask materials, to identify generalized patterns of aerosol exposure through leak points in the mask. We furthermore focus on patterns associated with simulated exhalation, to understand how persons around the subject might come into contact with potentially infectious aerosols, rather than investigating how the facemask-wearer might be protected from aerosol exposure. Where the box environment permitted a greater degree of control over the sampling conditions, the open bench environment yielded insight into the performance of these treatments in more realistic environments like what may be found in the workplace and in ambient conditions similar to other studies [39, 40]. By using a 3D printed replica, this strategy establishes a high-throughput and personalizable platform for elucidating the expected performance of a range of facemask materials and types. Based on the background measurements collected before conducting the experiments, the limits of detection for the six particle size bins are as follows: >2400 counts in the 0.3–0.5 μm size bin; >67 counts in the 0.5–1 μm size bin; >22 counts in the 1–3 μm size bin; >11 counts in the 3–5 μm size bin; >6 counts in the 5–10 μm size bin; and >5 counts in the >10 μm size bin. These values reflect the greatest variability between all of the sampling conditions. The particle counter used in this study is able to detect single particles at an efficiency of 100% for those of diameter >0.45μm, up to 2.1 x 108 counts/m^3^. For more information regarding the device and operating conditions, the reader is referred to the experimental section. Despite noise from ambient aerosols in both environments, somewhat variable output from the nebulizer, and some inconsistency in particle stream locations, we observed similar results for the facemask treatments, with facemasks tending to decrease particle levels in front of the replica but potentially redirecting a fraction of aerosol trajectories, increasing the potential for transmission at different vulnerable locations around the facemask and replica. This result quantitatively represents results observed by Kähler and Hain, who demonstrated that gaps in materials can lead to less effective filtration [44]. Under both the enclosed box and open bench conditions, the best-performing facemasks based on lowest aerosol transmission were N95T and the SewnF, with the N95T transmitting <5% of particles in the measured size bins. This evaluation is supported by the likelihood of observing reduced particle counts by random variation, as determined by Tukey’s Honest Significant Difference post-hoc tests in the enclosed box experiments, where the N95T was significantly different in all positions, and the SewnF was different in all particle sizes but the 5–10 μm size bin. This result differs from previous studies that had found that surgical masks were generally superior to cotton, homemade counterparts, though these studies do not sample using 3D printed models and use different particles for measurement [28, 45, 46]. Depending on the conformation of each facemask relative to the face geometry, gaps resulted from fitting the facemask, and these gaps opened low-pressure outlets for the redirection of particles, as easily visualized by the change in bar height between facemask types across the rows of **Fig 3** and vulnerability locations in **Fig 5**. Cloth facemasks permit more particles to exit at the top of the facemask on our face model, but this effect could be mitigated through the use of a nose clip or filter. However, the limit of nose clip effectiveness is evidenced by the Surgical mask results, which highlight the potential for particle redirection to the sides of the facemask and further emphasize fit-dependence of particle transmission. This is a potential limitation of more rigid facemasks that are unable to conform to facial features. Overall, our results support that facemasks aid in blocking exhaled particulates directly in front of the wearer and further solidify the importance of using facemask designs that conform to the individual wearer’s facial features to minimize the presence and extent of gaps, which cause vulnerabilities.

When particles were redirected out of gaps in the facemask, the particle size distribution shifted to larger particle sizes (see **Fig 6**), suggesting the possibility of a secondary benefit of facemask use: more rapid settling of exhaled aerosols to aid viral transmission reduction. Larger particles settle out of air faster than smaller ones, indicating that not only do facemasks reduce the overall aerosol exhaled output, but the aerosols that *do* escape through facemask gaps will likely remain in the air for shorter periods of time than those from unmasked counterparts, which would be expected to lower their potential for viral transmission [7, 47–49]. A decrease in submicron particulate count and an increase in overall CMD of escaped aerosols suggests that smaller particles experience either enhanced capture by the facemask material, aggregation or growth phenomena because of the facemask, or both phenomena simultaneously [7, 50]. As a reference, the CMD for the No Mask, Front position was 0.74 μm and fell in the 0.5–1 μm size bin. In contrast, the CMD for each of the Sewn, SewnPC, SewnF, N95L and Surgical facemasks fell in the 1–3 μm size bin (**Table 1** in text and **Table A in S1 File**) regardless of sampling location at the one-foot (0.3 m) distance, and, for all cases, the 0.3–0.5 μm and 0.5–1 μm bins were statistically different from the maximum reference (No Mask, Front). Natural respiratory emissions have been shown to have a similar size range as the output of the PARI nebulizer used in this study but in concentrations potentially orders of magnitude lower [47–49]. Humid environments and increased residence time within the facemask may support aggregation as well as hygroscopic growth, making aggregation a plausible explanation for reductions in particle counts and shift in CMD [51], especially at the aerosol concentrations tested. It is reasonable to assume that natural respiratory emissions will have interfacial properties that behave like the water-based aerosols in this study and could experience similar size distribution changes, which will require future validation with human studies. The aggregation analysis comparing aerosol volume further supports the hypothesis that aerosols may undergo aggregation or growth inside the humid mask environment, where smaller particles decrease by volume or frequency and mid-size particles increase by volume. This result is relevant to COVID-19 spread, which has been demonstrated to be enhanced in low ambient humidity, aligning with our claim that facemask-induced humidity and aerosol growth could lead to reduced viral transmission [52]. However, while it is identifiable by counts that smaller particles decrease in frequency, it is not readily apparent that mid-size particles relatively increase based solely on counts, since overall counts are lower with facemask use. The fold changes for facemasks indicate that there are significantly fewer particles overall for masked conditions compared to No Mask data, which makes it difficult to definitively determine whether particles are filtered out by the facemasks or are aggregating within the facemasks and becoming larger particles [50]. Finally, whether equivalently significant shifts in increased aerosol distributions occur in human respiratory emissions, or if this shift is enough to significantly impact the aerosol residence time surrounding the wearer, remains to be investigated.

From the evaluation of aerosol transmission as a function of distance (**Fig 4**), it was found that wearing a facemask significantly reduced the level of detectable aerosols at distances greater than one foot (0.3 m) from the model. The N95L, SewnF mask, and the Surgical mask produced particle counts above background one foot (0.3 m) away from the model, suggesting that person-to-person interactions at this distance could lead to unforeseen transmission of particles, obviating the critical need to evaluate multiple positions when quantifying facemask efficiency and supporting the need for social distancing and adequate surrounding ventilation. Unlike at the one-foot (0.3 m) distance, most facemasks were approximately at background levels at both the three- and six-foot (0.9 m and 1.8 m) distances with the exception of the previously mentioned N95T respirator, which is below background. All facemasks aside from the N95T respirator had particle counts mostly in the range of 4-5-fold above background, compared to 48-fold above background for measurements taken within one foot (0.3 m) of the facemask. This demonstrates the importance of observing the recommended greater than six-foot (1.8 m) social distancing guidelines to ensure particle transmission is minimized. The importance of social distancing can also be noted from the six-foot (1.8 m) distance background data, which changes very minimally over time. After almost three and a half hours of effectively continuous particle generation, the change in background indicates there was accumulation of 0.55 particles per second of the over 20,000 particles per second generated by the nebulizer. This indicates that even after prolonged exposure at the recommended six-foot (1.8 m) distance, there will be almost no increase in particle exposure under the background ambient air conditions observed in our experiment. It is important to note that the emitted aerosols may fall below our experimental detection limit and that computational models of indoor airflow have demonstrated that aerosol transmission can occur at distances greater than the six-foot (1.8 m) guidelines under certain background airflows and accumulation in enclosed spaces [10, 27].

While all other facemasks had particle counts at approximately background levels, the N95T had even lower particle that were below background; though the N95 did not have the largest pressure drop across the material, it was expected that the N95T respirator would have the lowest particle counts because of the performance, design, and fit of N95 respirators, which was shown to be the case in **Fig 7**. The N95L respirator, as expected, had greater particle counts than the N95T respirators, suggesting that the pressure drop across the material is not as significant of a factor as the fit of the facemask. As is shown in **Fig 4**, the N95T respirator showed counts reduced below background levels. This could theoretically be achieved by air flow patterns varying for this facemask in comparison to other facemasks since that there are fewer gaps, causing air to flow in directions away from the model. This also may point to the overall background noise from ambient aerosols and the limitations of our experimental sensitivity. While this observation was unexpected, it also symbolizes the difference in functionality that having a well fitted facemask can produce. The N95T did not exhibit a significant vulnerability and demonstrated escaped CMDs of < 0.5 μm, further indicating its effectiveness in filtering all but the smallest particles. The results of the vulnerability particle counting experiments further highlight the importance of fit with regard to the efficacy of facemask use, as particle counts at its vulnerability position were also at sub-background levels (**Fig 5**). While the Front position was typically well-protected from aerosolized droplets for most masks, other positions may be more vulnerable to escape of particles, especially if facemasks are not worn or fit properly. For example, the sewn masks, which fit tightly around the face model thanks to the straps tied around the back of the head of the model, outperformed the Surgical and N95L masks, indicating that even medical-grade protective equipment is not as effective if not worn properly.

In addition to evaluation of facemask vulnerabilities with regard to efficiency, we also evaluated the role of pressure drop in relation to particle transmission. Though made of different materials, the closeness in pressure-drop values between the Sewn mask and SewnPC mask was expected since these were nearly identical facemasks, sewn by the same person with similar materials (**Table 2**). Importantly, the N95 pressure-drop value represents both the N95T and N95L respirators. Values for pressure drop reported in literature are often performed using a TSI Automated Filter Tester Model 8130 and operated at 85 L/min; however, lower flow rates from the nebulizer used in our experiments necessitated measuring pressure drop across facemasks without the automated filter tester and at lower flow rates, which were measured to be 17 L/min (see **Supplemental Methods in S1 File**). This led to higher pressure drops than reported for N95 respirators, which are typically reported to have a pressure drop of 35 mm H_2_O or 0.3432 kPa [53]. The ability to test all facemasks with the same experimental conditions was crucial to the continuity of data and allowed for a homogeneous analysis of pressure-driven trends. The SewnF mask had the highest pressure drop by far, as was expected based on the use of a paper coffee filter, which had a large pressure drop when measured in the same experimental setup as the facemasks; the coffee filters had a pressure drop of 3.07 kPa and are thus thought to be the most significant factor in the pressure drop measurement from the SewnF mask. Somewhat unexpectedly, no obvious trend emerged relating pressure drop and facemask fit (**Fig 7**) unlike in previous studies that tested many materials, but did not account for particle size or location [54]. The N95L and N95T respirators exhibited strikingly different fit behaviors that were independent of their equivalent pressure drops. The lack of trends was further supported by observed particle counts for the respective sewn masks. The SewnF mask, which has pressure drops double or triple that of the other facemasks, exhibited overall particle counts similar to those of the N95T and N95L respirators. The Sewn mask had higher particle counts than the SewnPC and the Surgical masks, though despite the three facemasks having similar pressure drops across their materials, supporting the conclusion that fit plays a greater role than pressure drop, as the nose clip addition aided in reducing particle counts. While there was somewhat of a downward trend in particle counts as the pressure drop across the facemask material increases, the presence of other factors suggests that the trend does not appear to be due to pressure drop itself. Instead, the location of greater particle counts (vulnerabilities) seems to vary more than total particle counts, and the total particle counts seem to be more related to the fit of the facemask than the pressure drop across the facemasks’ respective materials.

Overall, this study illuminates significant areas previously overlooked in facemask research, including non-disruptive, positional sampling during facemask evaluation with a combined high-throughput face and airway model. Additionally, the use of water droplets in the size range of exhaled particles is critically important for ensuring that data will better reflect *in vivo* results. The identification of facemask vulnerabilities and their reduction in facemask efficiency relative to the Front location will hopefully influence future facemask research to further consider positional variability. Though our *in vitro* model is useful in evaluation of facemask effectiveness, it is limited in a few aspects, which are highlighted in **Table 3**. For example, there were particles exhaled outside of the range of 0.3–10 μm which are likely also capable of carrying viral copies and were not measured in this study because of the size range limitations of the particle counter used [3]. Additionally, while we used a face and airway model, we only implemented one face model, which was idealized to ensure broad applicability. Thus, the exact results reported here may not hold true for all face types, though we expect the trends to hold, particularly regarding the general importance of fit and particle counts being reduced as a function of distance. An advantage of using a 3D printed face model, however, is the potential to evaluate a multitude of faces, enabling researchers to increase the variability and sample size of faces used to better ensure that results would translate to the general public. For this study, a face model was used that has features within the average of a healthy adult male [55, 56]. Furthermore, while the exhaled water droplets mimicked those exhaled *in vivo*, the exhalation was imperfect; exhalation was at 17 L/min, which is low for adults, and breathing was achieved linearly through the mouth at ambient temperatures that do not mimic that of human breath. An additional limitation of the face-airway model is that, due to the construction based on averaged models, these parts do not align perfectly and there is an extension of the mouth airway model past the face model, potentially leading to an overestimate of the ability for a mask to reduce particle transmission. In future work, using a more realistic breathing profile with controlled temperature mimicking profiles during breathing and including nasal exhalation would improve the comparison to *in vivo* data. Future studies could also benefit from the use of more biologically relevant particles than pure water, such as mucin-containing phosphate buffered saline, due to studies detailing that the composition of disease-carrying aerosols is complex and includes multitudes of proteins, salts, bacteria, and viruses in water simultaneously [57–59]. Additionally, the use of lower particle concentrations would better mimic *in vivo* exhalation. Finally, including more facemask designs and materials would allow for even more robust analysis and guidance on ideal facemask design. Notably, the N95 respirator would benefit from evaluation of fit according to OSHA standards on a matching face model. These limitations are considered to be the most pertinent for future work; however, the identification of vulnerabilities, support of social distancing, affirmation of the importance of mask use in reducing particle counts, and determination of overall trends in results are not expected to vary even with these potential improvements.

**Table 3:** Overview of limitations and benefits of this study.

Benefits	Limitations
Model includes face and airways	Particles <0.3 μm and >10 μm not measured
Approach translatable to other face models, masks, etc.	Single, highly idealized model
Clinically relevant particle sizes	Exhalation non-cyclic, mouth only, single low flow rate without temperature control
3D printing allows for personalization	High aerosol concentration
Broad range of positions evaluated	N95T unable to undergo OSHA standard fit testing

The facemasks evaluated in this work are currently particularly relevant due to the global pandemic caused by COVID-19; researchers have used the efficiency of a facemask to estimate viral transmission from an infected, masked individual occupying a poorly ventilated room to a person briefly visiting said room. To replicate this for comparative purposes, calculations were performed based on reasonable assumptions of breathing patterns and viral copy concentrations, as reported in the **Supplemental Methods** Confined Area Viral Copy Exposure Calculations in **S1 File**. Based on the data of aerosol escape from a facemask gap, a second unmasked visitor to the room would be expected to inhale as many as 400,000 to 2 million viral copies in a 10-minute period. This stands in contrast to previous estimations that assume facemask filtration efficiencies as high as 99% and predict fewer than 75 viral copies. While the exact number of viral copies needed to cause infection has yet to be determined, our work identifies key limitations of previous research, which underestimates the number of aerosols that could be carrying viral copies and thus may lead to lax public policy guidelines, especially for indoor locations. Our research indicates that a combination of factors, including facemask fit, vulnerability locations, and distance from the face all contribute to the efficacy of a facemask in preventing exhaled particle transmission; efficacy for a facemask material might remain at 99% while the mask itself has an efficacy of an order of magnitude lower due to poor fit. This calculation is not intended to estimate viral transfer but instead to illuminate shortcomings in assumptions of facemask efficiencies that neglect fit, distance, and other relevant parameters. These variabilities can drastically influence guidance on best facemask practices and social distancing, and it is crucial that these considerations are incorporated into future facemask research.

## Conclusions

The results of this study suggest a number of factors that are important for consideration regarding the use of facemasks. The most critical suggestion from this study is that the use of facemasks, even if improperly fitted, is superior to not wearing a facemask in the reduction in the transmission of exhaled particulates, which are the primary mode of infection for COVID-19 and other respiratory illnesses. Furthermore, our study serves as an improvement on facemask evaluation, utilizing non-invasive determination of particle transmission from a number of facemask types at multiple locations around a face. Though not finalized guidance on the best possible facemask, our results do demonstrate that certain facemask features can reduce particle counts. For example, of the sewn facemasks, the facemask with a coffee filter in between layers of cotton fabric produced the lowest particle counts. Notably, including a pipe cleaner over the nose also reduced particle counts. However, these reduced counts are seen in vulnerable areas and not in front of the facemask; thus, studies which only measure aerosol counts in front of a facemask would be misleading. Of the facemasks tested, the N95T respirator was the best-performing facemask, the N95L, Surgical, and sewn masks all allowed 35-70-fold more particles to escape relative to the N95T, suggesting that medical-grade, manufactured facemasks should not be a priority for the general population and should instead be prioritized for healthcare workers who will be fit-tested properly. Previous studies have focused more on pressure drop across materials and breathability of materials; while we have found that there is somewhat of a decrease in particle counts as the pressure drop across a material increases, there is a greater difference in particle counts in redirected areas of the facemask (vulnerabilities) compared to particle count reduction due to pressure drop. This suggests that fit and air flow are important factors to consider when evaluating best facemask practices for reducing particle counts and are likely more important factors than pressure drop. Even in cases of poor fit, our results suggest that facemasks will still reduce the overall transmission of aerosols from the wearer. Our results suggest that poorly fitted facemasks will still reduce the overall number of transmission aerosols directly in front of the wearer and may promote aerosol growth that would be further beneficial for reducing transmission. However, based on our estimations of exposure, poorly fitting facemasks can still result in significant aerosol escape that can result in accumulation in unventilated areas that may still promote transmission. This further supports that facemask usage is most effective in conjunction with social distancing and good ventilation to prevent aerosol accumulation and transmission potential. Finally, with these recommendations, we also note that facemask fit is an important parameter to minimize transmission of droplets and aerosols, which is true both for medical facemasks and homemade sewn facemasks.

## Supporting information

S1 Dataset Dataset. Collected aerosol distribution data.
(ZIP)

S1 File Supplemental information. This file contains supplemental methods, figures, tables, and references.
(DOCX)

S2 File Box comparisons by aerosol size. This file contains statistical analyses of the box data, along with interactive versions of the 95% confidence intervals for estimated marginal means and pairwise p-value plots.
(HTML)

S3 File Bench comparisons by aerosol size. This file contains statistical analyses of the bench data at 1 ft, 3 ft, and 6 ft distances (0.3 m, 0.9 m, and 1.8 m), along with interactive versions of the 95% confidence intervals for estimated marginal means and pairwise p-value plots.
(HTML)

S4 File Contour plots by aerosol size. Contour plots of background-subtracted aerosol counts taken at 1 ft, 3 ft, and 6 ft distances (0.3 m, 0.9 m, and 1.8 m).
(HTML)

S1 Video No mask experimental setup. This video is depicting a bench experiment with No Mask counting particles in the front position. Visible are the face and airway model, nebulizer, and particle counter. The apparent mist is the water particles generated by the nebulizer.
(MP4)

S2 Video Sewn mask experimental setup. This video is depicting a bench experiment with the Sewn mask counting particles in the front position. Visible are the face and airway model covered by the Sewn mask, as well as the nebulizer, and particle counter. The apparent mist is the water particles generated by the nebulizer, which is noticeably transmitting particles through the front of the mask and gaps at the sides and top of the mask.
(MP4)

S3 Video Surgical mask experimental setup. This video is depicting a bench experiment with the Surgical mask counting particles in the front position. Visible are the face and airway model covered by the Surgical mask, as well as the nebulizer, and particle counter. The apparent mist is the water particles generated by the nebulizer, which is noticeably transmitting particles through the gaps at the sides and top of the mask.
(MP4)
