# Supplementary material for: Check the gap: Facemask performance and exhaled aerosol distributions around the wearer
Source: PLoS One. 2020 Dec 16;15(12):e0243885. doi: 10.1371/journal.pone.0243885 (PMC7744055; doi:10.1371/journal.pone.0243885)
Supplement: S1 File — This file contains supplemental methods, figures, tables, and references. (DOCX) [file pone.0243885.s002.docx]

##

**Supplemental Information**

Check the Gap: Facemask Performance and Exhaled Aerosol Distributions Around the Wearer

Emily L. Kolewe^1¶^, Zachary Stillman^1¶^, Ian R. Woodward^1¶^, and Catherine A. Fromen, PhD^1*^

^1^ Department of Chemical and Biomolecular Engineering, University of Delaware, Newark, Delaware, United States of America

* Corresponding author

E-mail: cfromen@udel.edu (CAF)

^¶^These authors contributed equally to this work.

[**Supplemental Methods** 3](#_Toc53740780)

[Aggregation Analysis 3](#_Toc53740781)

[Confined Area Viral Copy Exposure Calculations 3](#_Toc53740782)

[Statistical Analysis 4](#_Toc53740783)

[Velocity Measurements 5](#_Toc53740784)

[Equipment Categorization Method 5](#_Toc53740785)

[Face Model Analysis 6](#_Toc53740786)

[**Supplemental Figures** 7](#_Toc53740787)

[Figure A. Face and Airway Model Design 7](#_Toc53740788)

[Figure B. Schematic overview of the pressure-drop experimental setup 8](#_Toc53740789)

[Figure C. Velocity Measurements Experimental Setup. 8](#_Toc53740790)

[Figure D. Background subtracted particle counts on the bench at distance of 1, 3, and 6 feet from the face model. 9](#_Toc53740791)

[**Supplemental Tables** 10](#_Toc53740792)

[Table A. Count Median Diameters (CMD) 10](#_Toc53740793)

[Table B. Pressure-drop statistics 10](#_Toc53740794)

[Table C. Parameters used to calculate visitor exposure. 10](#_Toc53740795)

[**Supplemental References** 12](#_Toc53740796)

# **Supplemental Methods**

## **Aggregation Analysis**

Particle counts (C) that were averaged between experimental replicates for a given particle diameter (D), mask (m), position (p), and distance (d), and having had background particle counts subtracted, were then translated into volumes based on the particle diameter and **SI Equation 1** below. The total of all particle volumes for a single experiment were calculated using **SI Equation 2**, and particle volumetric fractions were calculated based on **SI Equation 3**. Relative volumetric fractions were calculated with respect to the case of No Mask used in the box experimental set up at a distance within 4 inches in the Front position, as is shown in **SI Equation 4**. Finally, by summing the relative volumetric fractions across all masks and dividing by the number of masks (M), the average relative volumetric fraction was reported, shown in **SI Equation 5**.

**SI** **Eq. 1**. $V_{D, m, p, d}= C_{D, m, p, d}*\frac{4}{3}\Pi\left( \frac{D}{2} \right)^{3}$
**SI Eq. 2**. ${Vtotal}_{m, p, d} = \sum_{D} V_{D, m, p, d}$
**SI Eq. 3**. ${Vfrac}_{D, m, p, d} = \frac{V_{D, m, p, d}}{{Vtotal}_{m, p, d}}$
**SI Eq. 4**. ${Vrelative}_{D, m, p, d} =\frac{{Vfrac}_{D, m, p, d}}{{Vfrac}_{D,No Mask, Front, 4 in}}$
**SI Eq. 5**. ${Vaverage,relative}_{D, p, d} = \frac{\sum_{m} {Vrelative}_{D, m, p, d}}{M}$

## **Confined Area Viral Copy Exposure Calculations**

To estimate the exposure of someone who walks briefly into a confined area, such as an unventilated room with closed windows and doors, in which another person has been in for a long period of time, a calculation was performed based on breathing metrics and what is currently known about COVID-19 exhaled particles. This calculation was intended to help illuminate potential hazards of the workplace. The parameters used and assumed during this calculation are described in **Table S4**. First, the number of viral copies exhaled by an individual in a room with a mask on was calculated based on the rate of breathing particles and the efficiency of a mask, in this initial calculation assumed to be 99% based on previous research, performed using **SI Equation 6**. This initial viral copy value was used in conjunction with the half life to calculate the number of viral copies remaining using **SI Equation 7**. This was translated to a concentration by dividing by volume in **SI Equation 8**. Finally, the exposure of a visitor breathing at a normal rate was calculated using **SI Equation 9**. While this value was calculated to be 75 copies, by changing the efficiency to reflect particles which escape through gaps and are transmitted by the wearer, the efficiency value ranging to even a modest 80% would increase the viral copy exposure of a visitor to over 1,500 copies in the same 10 minute period of time. Viral copy exposure to be as high as 200,000 at low mask efficiencies, such as with a poorly fit mask.

**SI Eq. 6** $N_{0}= {VC}_{exhaled} * V * (1-\epsilon)$

**SI Eq. 7** $N(t) =N_{0}{\frac{1}{2}}^{\left( \frac{T_{i}}{t_{1/2}} \right)}$

**SI Eq. 8** $VC = \frac{N}{V}$

**SI Eq. 9** $N_{exposure}= B * VC * T_{e}$

## **Statistical Analysis**

To navigate the challenge of negative background-subtracted counts interfering with log transformation, untransformed data were used for statistical analysis.

*Box Experiments*

Statistical analyses were conducted by performing a 2-factor multivariate analysis of variance (MANOVA) on the background-subtracted particle counts, using the multivariate response based on particle size bin for the two factors of facemask treatment and sample location around the face. Subsequent 2-factor ANOVA procedures were performed for each particle size bin. Tukey’s Honest Significant Difference test for multiple comparisons was performed using the *emmeans* package, and the results were plotted as 95% confidence intervals and pairwise p-value plots for the estimated marginal means.

*Bench Experiments*

Statistical analyses were conducted by performing a 3-factor multivariate analysis of variance (MANOVA) on the background-subtracted particle counts, using the multivariate response based on particle size bin for the three factors of facemask treatment, counter location, and sample location around the face. Subsequent 3-factor ANOVA procedures were performed for each particle size bin. Tukey’s Honest Significant Difference test for multiple comparisons was performed using the *emmeans* package, and the results were plotted as 95% confidence intervals and pairwise p-value plots for the estimated marginal means.

For a discussion on interpretation of the plots produced by the emmeans package, please refer to this vignette: <https://cran.r-project.org/web/packages/emmeans/vignettes/comparisons.html>

## **Velocity Measurements**

Velocity was measured using a Testo 405i hot wire anemometer handheld probe (Testo Inc., Titisee-Neustadt, Germany) at the exit of the face model mouth with the nebulizer running, as is shown in **Fig S2**. Measurements were taken in triplicate and flow rate was calculated using the diameter of the idealized mouth and **SI Equation S10**:

**SI Eq. 10** $Q = v * \Pi\left( \frac{D}{2} \right)^{2}$

With the velocity measured to be 0.9 m/s and the diameter of the mouth being 20 mm or 0.02 m, the flow rate exiting the mouth was calculated to be 2.83 E-4 m^3^/s or 17 L/min.

## **Equipment Categorization Method**

There are many potential methods to categorization equipment which covers the mouth and/or nose with the intention of reducing the spread of airborne particulates. There are four centers/organizations (FDA, WHO, CDC, and NHS) with (a) brief descriptions of their categorization methods and (b) a reference, followed by a summary.

1. The Food and Drug Administration (FDA) describes a respirator (N95) and surgical mask in separate categories from face masks, but a face mask can include “cloth face coverings” as a subcategory (but this is not defined). These are all separate from face shields. It is not clear how a bandana or other equipment fits into this categorization, but it seems as though they would be in another separate, undefined category of their own.^1^

2. The World Health Organization (WHO) defines respirators (N95s), medical masks (surgical, procedural, and other cloth masks), and face shields all separately, but face mask is used very loosely to mean all of the above. There is no definition of face coverings.^2^

3. The Center for Disease Control (CDC) defines face shield, surgical mask, and respirator all separately. The words “face cover” are in the html and the video title, but not in the actual document and are not defined. Also, ‘face mask’ is not defined but is stated to be different from a surgical mask.^3^

4. The National Health Service (NHS) distinguishes face masks from face coverings. Face masks are medical devices (respirators [N95s] or medical masks [surgical, procedural, etc]) and face coverings are everything else (homemade/store bought non-medical masks, bandanas, face shields).^4^

Summary

1. Homemade or store bought sewn masks can be categorized as a facemask (FDA, WHO) or not (CDC, NHS)
2. Respirators and surgical masks are not the same, but both can be called facemasks (WHO, NHS)
3. Face coverings are not well defined, seem to be less ‘official’, and seem to not include surgical masks or N95s (CDC, FDA, NHS)

## **Face Model Analysis**

A face model was selected such that the nose, mouths, ears, chin, and neck were all available to ensure facemask fit would be representative of a human subject. The model selected was then analyzed to ensure that the face was within reasonable measurements of an average adult male (see **Fig S1**) while also being as small as possible to ensure the ability to 3D print the model. Additionally, the nose and mouth models were connected to the face and thus were aligned manually using the aligning feature in Autodesk MeshMixer.

# **Supplemental Figures**


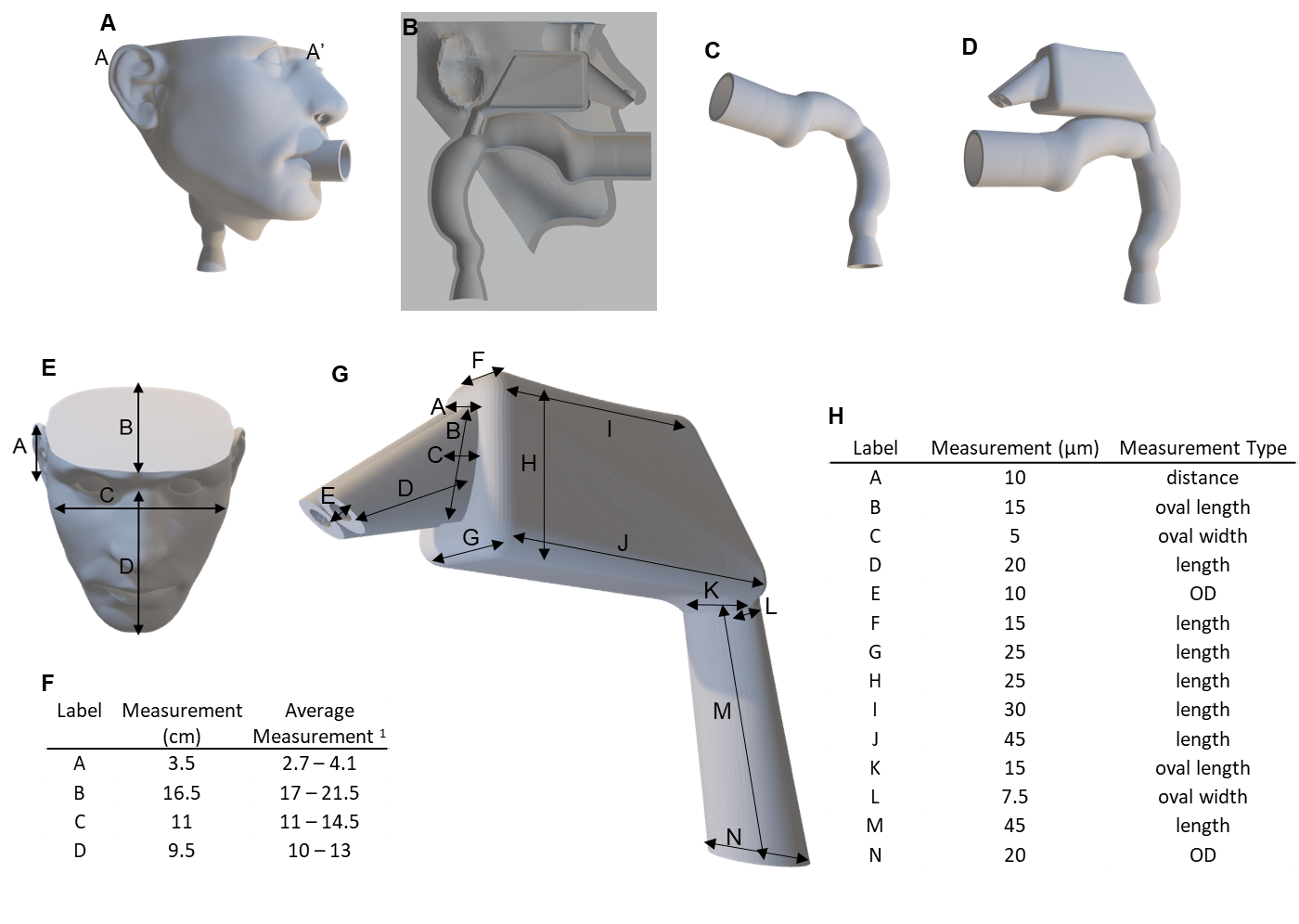


## **Figure A. Face and Airway Model Design**

A) The face and airway model showing the full face and a cut view (A A’) which allows for visibility of the alignment of the mouth and nasal passages to the face model in B. C) An idealized mouth and upper throat, representative of healthy adults, and the connection to an idealized nasal passage in D. E) The face model with indicators for measurements taken, corresponding to the measurement values listed in the table in F. G) An idealized nasal passage based on an averaged model, the dimensions of which are described in the table in H.


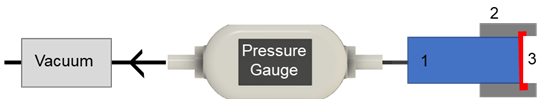


## **Figure B. Schematic overview of the pressure-drop experimental setup**

This schematic depicts the vacuum and pressure gauge to generate and measure air flow, as well as 1. a holder (blue), 2. a cap (gray), and 3. a mask material (red) sandwiched between the holder and cap.


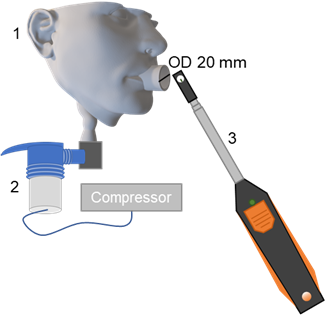


## **Figure C. Velocity Measurements Experimental Setup.**

Velocity measurements were taken at the outlet of the face model (1) with the nebulizer (2) connected to the compressor. Velocity was measured using an anemometer (3).


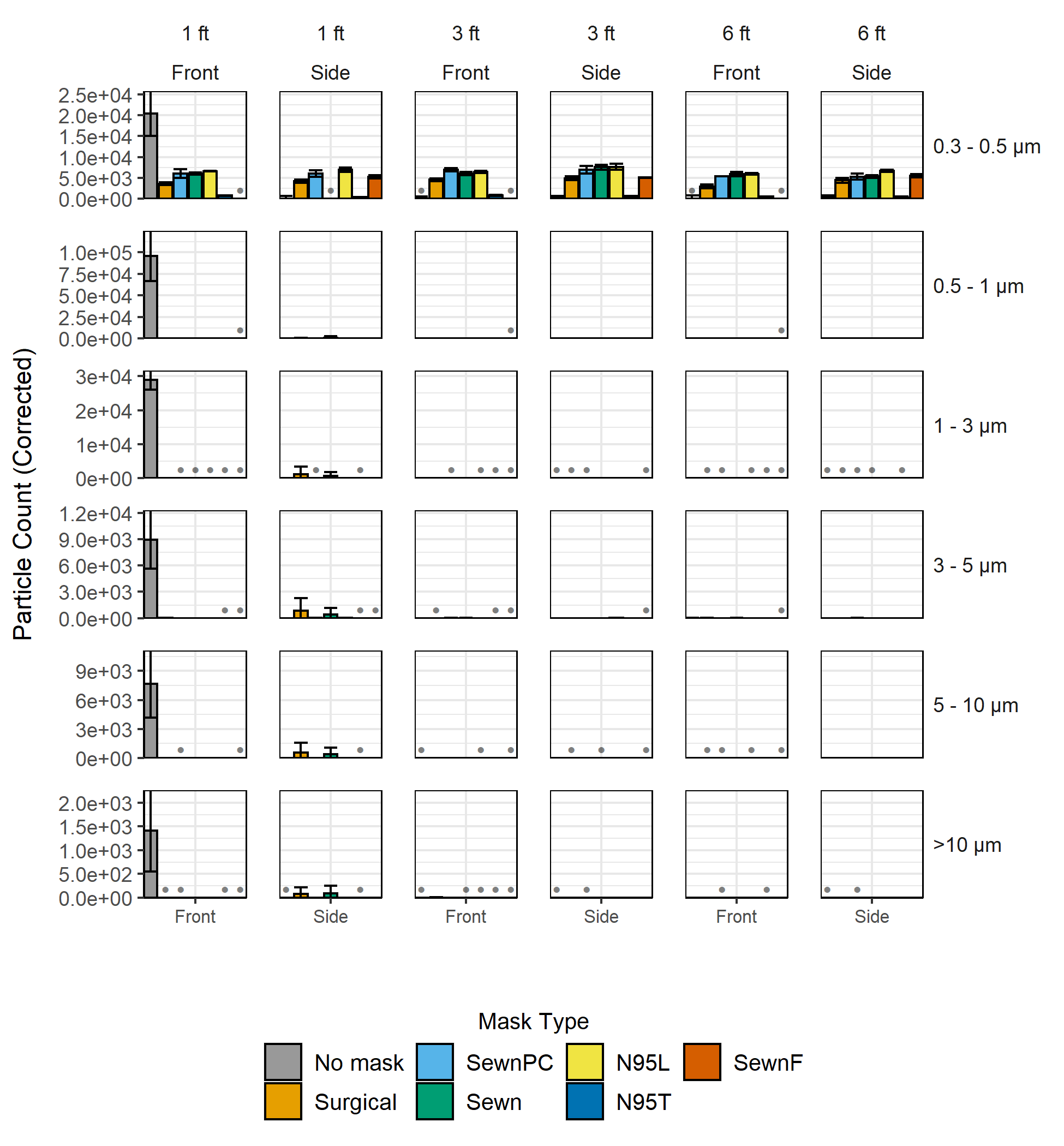


## **Figure D. Background subtracted particle counts on the bench at distance of 1, 3, and 6 feet from the face model.**

# **Supplemental Tables**

## **Table A. Count Median Diameters (CMD)**

Count Median Diameters (CMD) of particles collected in varied locations between facemask treatments within the enclosed box. CMD in μm shown for each location and grouped by facemask type, including No Mask, Sewn, SewnF, SewnPC, Surgical, N95L and N95T.

|  | **Front** | **Side** | **Top** |
| --- | --- | --- | --- |
| No Mask | 0.74 | - | - |
| Surgical | 1.49 | 1.85 | 0.80 |
| Sewn | 3.33 | 4.00 | 1.84 |
| SewnF | 2.96 | - | 2.54 |
| SewnPC | 2.78 | - | 2.46 |
| N95L | 2.32 | 3.84 | - |
| N95T |  | - | <0.5 |

## **Table B. Pressure-drop statistics**

Pressure-drop and standard deviation for the mask material; this table is a continuation of Table 2. Notably, there are mask materials with a standard deviation of 0 due to the pressure readings being equal within the range of error of the pressure gauge used.

| Facemask | Surgical | SewnPC | Sewn | N95 | SewnF |
| --- | --- | --- | --- | --- | --- |
| Pressure Drop (kPa) | 0.5 | 0.63 | 1.2 | 1.4 | 3.07 |
| Standard Deviation | 0 | 0.058 | 0 | 0 | 0.058 |

## **Table C. Parameters used to calculate visitor exposure.**

The parameters described below were used in **SI Methods Confined Area Viral Copy Exposure Calculations** in **SI Equations 6 - 9** to calculate the exposure to viral copies of a visitor to a room that had previously been occupied by a person wearing a mask in order to better understand the risks of realistic work- or school-like environments.

| Parameter | Parameter Value | Units | Symbol |
| --- | --- | --- | --- |
| virus copies exhaled | 100 | Copies/L | ${VC}_{exhaled}$ |
| Breathing rate | 15 | breaths/min at 1L | *B* |
| Room volume | 40,000 | L | *V* |
| Time person is sitting in a room | 120 | min | $T_{i}$ |
| Halftime of virus in air | 125 | min | $t_{1/2}$ |
| filtration efficiency | 0.99 | unitless | $\epsilon$ |
| virus copies exhaled into room | 20562.28 | Copies | *N* |
| virus copies/L exhaled into room | 0.514057 | Copies/L | *VC* |
| Duration of new person in room | 10 | min | $T_{e}$ |
| Inhaled viral copies by visitor in 10 min | 77.10854 | Copies | $N_{exposure}$ |

# **Supplemental References**

1. Hinton, D. M. In *Letter from Denise M. Hinton to Manufacturers of Imported, Non-NIOSH-Approved Disposable Filtering Facepiece Respirators; Health Care Personnel; Hospital Purchasing Departments and Distributors; Importers and Commercial Wholesalers; and Any Other Applicable Stakeholders, March 28, 2020*, United States. Food and Drug Administration, United States. Food and Drug Administration: 2020.

2. Organization, W. H. *Advice on the use of masks in the context of COVID-19: interim guidance, 5 June 2020*; World Health Organization: 2020.

3. Prevention, C. C. f. D. C. a., Coronavirus Disease 2019 (COVID-19). <https://www.cdc.gov/coronavirus/2019-ncov/prevent-getting-sick/cloth-face-cover-guidance.html>, 2020.

4. Wilson, a., Face covering vs face mask: What is the difference? *Express* 2020.
