# Supplementary material for: Check the gap: Facemask performance and exhaled aerosol distributions around the wearer
Source: PLoS One. 2020 Dec 16;15(12):e0243885. doi: 10.1371/journal.pone.0243885 (PMC7744055; doi:10.1371/journal.pone.0243885)
Supplement: S2 File — This file contains statistical analyses of the box data, along with interactive versions of the 95% confidence intervals for estimated marginal means and pairwise p-value plots. (HTML) [file pone.0243885.s003.html]

Check the Gap: Facemask Performance and Exhaled Aerosol Distributions Around the Wearer


# Check the Gap: Facemask Performance and Exhaled Aerosol Distributions Around the Wearer

#### Emily L. Kolewe,☨ Zachary S. Stillman,☨ Ian R. Woodward,☨ and Catherine A. Fromen, Ph.D.

Department of Chemical and Biomolecular Engineering, University of Delaware, Newark, DE 19716  
☨Contributed Equally  
\*Corresponding Author  
cfromen@udel.edu

#### 2020-09-01 (Last compiled)


## Box Comparisons by Aerosol Size

### MANOVA Output

```
##              Df Pillai approx F num Df den Df    Pr(>F)    
## Mask          6 2.2434   4.1804     36    252 7.609e-12 ***
## ExpCode       2 1.3429  12.9423     12     76 5.997e-14 ***
## Mask:ExpCode 12 2.8238   3.1117     72    252 2.434e-11 ***
## Residuals    42                                            
## ---
## Signif. codes:  0 '***' 0.001 '**' 0.01 '*' 0.05 '.' 0.1 ' ' 1
```

```
##  Response 1 :
##              Df    Sum Sq  Mean Sq F value    Pr(>F)    
## Mask          6 443703067 73950511  27.411 4.921e-13 ***
## ExpCode       2  85727673 42863836  15.888 7.277e-06 ***
## Mask:ExpCode 12 854921091 71243424  26.408 9.886e-16 ***
## Residuals    42 113309249  2697839                      
## ---
## Signif. codes:  0 '***' 0.001 '**' 0.01 '*' 0.05 '.' 0.1 ' ' 1
## 
##  Response 2 :
##              Df     Sum Sq   Mean Sq F value    Pr(>F)    
## Mask          6 1726645382 287774230 10.8000 3.040e-07 ***
## ExpCode       2  472722322 236361161  8.8705 0.0006117 ***
## Mask:ExpCode 12 4457339761 371444980 13.9401 5.125e-11 ***
## Residuals    42 1119122809  26645781                      
## ---
## Signif. codes:  0 '***' 0.001 '**' 0.01 '*' 0.05 '.' 0.1 ' ' 1
## 
##  Response 3 :
##              Df     Sum Sq   Mean Sq F value    Pr(>F)    
## Mask          6  239752154  39958692  4.4932  0.001338 ** 
## ExpCode       2  116635145  58317572  6.5575  0.003323 ** 
## Mask:ExpCode 12 1226985319 102248777 11.4974 1.002e-09 ***
## Residuals    42  373515571   8893228                      
## ---
## Signif. codes:  0 '***' 0.001 '**' 0.01 '*' 0.05 '.' 0.1 ' ' 1
## 
##  Response 4 :
##              Df    Sum Sq  Mean Sq F value    Pr(>F)    
## Mask          6  47713985  7952331  6.9835 3.378e-05 ***
## ExpCode       2  30091612 15045806 13.2127 3.537e-05 ***
## Mask:ExpCode 12 230015114 19167926 16.8327 2.410e-12 ***
## Residuals    42  47826833  1138734                      
## ---
## Signif. codes:  0 '***' 0.001 '**' 0.01 '*' 0.05 '.' 0.1 ' ' 1
## 
##  Response 5 :
##              Df   Sum Sq Mean Sq F value   Pr(>F)    
## Mask          6 15627268 2604545  4.5653 0.001193 ** 
## ExpCode       2  8672216 4336108  7.6003 0.001523 ** 
## Mask:ExpCode 12 84826556 7068880 12.3904 3.22e-10 ***
## Residuals    42 23961610  570515                     
## ---
## Signif. codes:  0 '***' 0.001 '**' 0.01 '*' 0.05 '.' 0.1 ' ' 1
## 
##  Response 6 :
##              Df  Sum Sq Mean Sq F value    Pr(>F)    
## Mask          6  187886   31314  2.7584   0.02370 *  
## ExpCode       2   67027   33514  2.9521   0.06315 .  
## Mask:ExpCode 12 1106917   92243  8.1255 1.367e-07 ***
## Residuals    42  476796   11352                      
## ---
## Signif. codes:  0 '***' 0.001 '**' 0.01 '*' 0.05 '.' 0.1 ' ' 1
```

### 0.3 - 0.5 μm

### 0.5 - 1 μm

### 1 - 3 μm

### 3 - 5 μm

### 5 - 10 μm

### > 10 μm
