# Supplementary material for: Check the gap: Facemask performance and exhaled aerosol distributions around the wearer
Source: PLoS One. 2020 Dec 16;15(12):e0243885. doi: 10.1371/journal.pone.0243885 (PMC7744055; doi:10.1371/journal.pone.0243885)
Supplement: S3 File — This file contains statistical analyses of the bench data at 1 ft, 3 ft, and 6 ft distances (0.3 m, 0.9 m, and 1.8 m), along with interactive versions of the 95% confidence intervals for estimated marginal means and pairwise p-value plots. (HTML) [file pone.0243885.s004.html]

Check the Gap: Facemask Performance and Exhaled Aerosol Distributions Around the Wearer


# Check the Gap: Facemask Performance and Exhaled Aerosol Distributions Around the Wearer

#### Emily L. Kolewe,☨ Zachary S. Stillman,☨ Ian R. Woodward,☨ and Catherine A. Fromen, Ph.D.

Department of Chemical and Biomolecular Engineering, University of Delaware, Newark, DE 19716  
☨Contributed Equally  
\*Corresponding Author  
cfromen@udel.edu

#### 2020-09-01 (Last compiled)


## Bench Comparisons by Aerosol Size

### MANOVA Output

```
##                                     Df  Pillai approx F num Df den Df    Pr(>F)
## Mask                                 6 2.08771      7.5     36    504 < 2.2e-16
## counter_location                     2 1.35012     27.7     12    160 < 2.2e-16
## face_location                        1 0.99909  14382.5      6     79 < 2.2e-16
## Mask:counter_location               12 2.05081      3.6     72    504 < 2.2e-16
## Mask:face_location                   6 2.01786      7.1     36    504 < 2.2e-16
## counter_location:face_location       2 1.00287     13.4     12    160 < 2.2e-16
## Mask:counter_location:face_location 12 2.03826      3.6     72    504 < 2.2e-16
## Residuals                           84                                         
##                                        
## Mask                                ***
## counter_location                    ***
## face_location                       ***
## Mask:counter_location               ***
## Mask:face_location                  ***
## counter_location:face_location      ***
## Mask:counter_location:face_location ***
## Residuals                              
## ---
## Signif. codes:  0 '***' 0.001 '**' 0.01 '*' 0.05 '.' 0.1 ' ' 1
```

```
##  Response 1 :
##                                     Df    Sum Sq  Mean Sq F value    Pr(>F)    
## Mask                                 6 468673005 78112167 81.4422 < 2.2e-16 ***
## counter_location                     2  14461428  7230714  7.5390 0.0009743 ***
## face_location                        1   8465809  8465809  8.8267 0.0038703 ** 
## Mask:counter_location               12 602257944 50188162 52.3278 < 2.2e-16 ***
## Mask:face_location                   6 384106000 64017667 66.7468 < 2.2e-16 ***
## counter_location:face_location       2 188807313 94403657 98.4282 < 2.2e-16 ***
## Mask:counter_location:face_location 12 467717473 38976456 40.6381 < 2.2e-16 ***
## Residuals                           84  80565371   959112                      
## ---
## Signif. codes:  0 '***' 0.001 '**' 0.01 '*' 0.05 '.' 0.1 ' ' 1
## 
##  Response 2 :
##                                     Df     Sum Sq   Mean Sq F value    Pr(>F)
## Mask                                 6 3859482285 643247047  31.850 < 2.2e-16
## counter_location                     2 1333773035 666886517  33.020 2.624e-11
## face_location                        1  631807746 631807746  31.283 2.720e-07
## Mask:counter_location               12 7843094157 653591180  32.362 < 2.2e-16
## Mask:face_location                   6 3950178301 658363050  32.598 < 2.2e-16
## counter_location:face_location       2 1289421947 644710974  31.922 4.874e-11
## Mask:counter_location:face_location 12 7886207442 657183953  32.540 < 2.2e-16
## Residuals                           84 1696490393  20196314                  
##                                        
## Mask                                ***
## counter_location                    ***
## face_location                       ***
## Mask:counter_location               ***
## Mask:face_location                  ***
## counter_location:face_location      ***
## Mask:counter_location:face_location ***
## Residuals                              
## ---
## Signif. codes:  0 '***' 0.001 '**' 0.01 '*' 0.05 '.' 0.1 ' ' 1
## 
##  Response 3 :
##                                     Df    Sum Sq  Mean Sq F value    Pr(>F)    
## Mask                                 6 349447892 58241315  181.13 < 2.2e-16 ***
## counter_location                     2 135049642 67524821  210.00 < 2.2e-16 ***
## face_location                        1  51663694 51663694  160.67 < 2.2e-16 ***
## Mask:counter_location               12 698479837 58206653  181.02 < 2.2e-16 ***
## Mask:face_location                   6 365244924 60874154  189.32 < 2.2e-16 ***
## counter_location:face_location       2 103594581 51797290  161.09 < 2.2e-16 ***
## Mask:counter_location:face_location 12 729300882 60775074  189.01 < 2.2e-16 ***
## Residuals                           84  27009962   321547                      
## ---
## Signif. codes:  0 '***' 0.001 '**' 0.01 '*' 0.05 '.' 0.1 ' ' 1
## 
##  Response 4 :
##                                     Df   Sum Sq Mean Sq F value    Pr(>F)    
## Mask                                 6 33052854 5508809  16.943 1.007e-12 ***
## counter_location                     2 14843450 7421725  22.827 1.210e-08 ***
## face_location                        1  4210469 4210469  12.950 0.0005397 ***
## Mask:counter_location               12 66057245 5504770  16.931 < 2.2e-16 ***
## Mask:face_location                   6 36244060 6040677  18.579 1.251e-13 ***
## counter_location:face_location       2  8458272 4229136  13.007 1.199e-05 ***
## Mask:counter_location:face_location 12 72432443 6036037  18.565 < 2.2e-16 ***
## Residuals                           84 27311197  325133                      
## ---
## Signif. codes:  0 '***' 0.001 '**' 0.01 '*' 0.05 '.' 0.1 ' ' 1
## 
##  Response 5 :
##                                     Df   Sum Sq Mean Sq F value    Pr(>F)    
## Mask                                 6 24152156 4025359 12.3695 6.199e-10 ***
## counter_location                     2 10608348 5304174 16.2991 1.044e-06 ***
## face_location                        1  3153764 3153764  9.6912 0.0025314 ** 
## Mask:counter_location               12 48323400 4026950 12.3744 4.698e-14 ***
## Mask:face_location                   6 26254594 4375766 13.4462 1.254e-10 ***
## counter_location:face_location       2  6359385 3179692  9.7708 0.0001531 ***
## Mask:counter_location:face_location 12 52554729 4379561 13.4579 5.509e-15 ***
## Residuals                           84 27335836  325427                      
## ---
## Signif. codes:  0 '***' 0.001 '**' 0.01 '*' 0.05 '.' 0.1 ' ' 1
## 
##  Response 6 :
##                                     Df  Sum Sq Mean Sq F value    Pr(>F)    
## Mask                                 6  819720  136620  7.4052 2.227e-06 ***
## counter_location                     2  353402  176701  9.5777 0.0001791 ***
## face_location                        1  108035  108035  5.8558 0.0176830 *  
## Mask:counter_location               12 1653354  137779  7.4680 3.594e-09 ***
## Mask:face_location                   6  895787  149298  8.0923 6.572e-07 ***
## counter_location:face_location       2  218537  109268  5.9226 0.0039243 ** 
## Mask:counter_location:face_location 12 1786347  148862  8.0687 7.752e-10 ***
## Residuals                           84 1549741   18449                      
## ---
## Signif. codes:  0 '***' 0.001 '**' 0.01 '*' 0.05 '.' 0.1 ' ' 1
```

### 0.3 - 0.5 μm

### 0.5 - 1 μm

### 1 - 3 μm

### 3 - 5 μm

### 5 - 10 μm

### > 10 μm
