# Supplementary material for: Check the gap: Facemask performance and exhaled aerosol distributions around the wearer
Source: PLoS One. 2020 Dec 16;15(12):e0243885. doi: 10.1371/journal.pone.0243885 (PMC7744055; doi:10.1371/journal.pone.0243885)
Supplement: S4 File — Contour plots of background-subtracted aerosol counts taken at 1 ft, 3 ft, and 6 ft distances (0.3 m, 0.9 m, and 1.8 m). (HTML) [file pone.0243885.s005.html]

Check the Gap: Facemask Performance and Exhaled Aerosol Distributions Around the Wearer


# Check the Gap: Facemask Performance and Exhaled Aerosol Distributions Around the Wearer

#### Emily L. Kolewe,☨ Zachary S. Stillman,☨ Ian R. Woodward,☨ and Catherine A. Fromen, Ph.D.

Department of Chemical and Biomolecular Engineering, University of Delaware, Newark, DE 19716  
☨Contributed Equally  
\*Corresponding Author  
cfromen@udel.edu

#### 2020-09-01 (Last compiled)


## Contour Plots by Aerosol Size

### Common Scales

### Individual Scales

### Individual Models and Plots

```
## [1] "No Mask 0.3"
##  [1] -50000      0   2000   4000   6000   8000  10000  12000  14000  16000
## [11]  18000  20000  22000  24000  26000
## 
## Call:
## lm(formula = background_subtracted ~ poly(r, degree = 2) + theta + 
##     r:theta, data = data_mask)
## 
## Residuals:
##    Min     1Q Median     3Q    Max 
##  -4236  -2554  -1495   1807   8328 
## 
## Coefficients:
##                      Estimate Std. Error t value Pr(>|t|)    
## (Intercept)            473.77    1341.15   0.353  0.72956    
## poly(r, degree = 2)1   537.89    5690.03   0.095  0.92613    
## poly(r, degree = 2)2 11841.24    4023.46   2.943  0.01142 *  
## theta                  211.90      40.16   5.276  0.00015 ***
## theta:r                -42.10      10.26  -4.105  0.00124 ** 
## ---
## Signif. codes:  0 '***' 0.001 '**' 0.01 '*' 0.05 '.' 0.1 ' ' 1
## 
## Residual standard error: 4023 on 13 degrees of freedom
## Multiple R-squared:  0.8025, Adjusted R-squared:  0.7417 
## F-statistic:  13.2 on 4 and 13 DF,  p-value: 0.0001641
```

```
## [1] "Surgical 0.3"
##  [1] -50000      0   2000   4000   6000   8000  10000  12000  14000  16000
## [11]  18000  20000  22000  24000  26000
## 
## Call:
## lm(formula = background_subtracted ~ poly(r, degree = 2) + theta + 
##     r:theta, data = data_mask)
## 
## Residuals:
##     Min      1Q  Median      3Q     Max 
## -592.70 -288.92   -2.51  226.52  710.28 
## 
## Coefficients:
##                       Estimate Std. Error t value Pr(>|t|)    
## (Intercept)           4503.750    152.129  29.605 2.57e-13 ***
## poly(r, degree = 2)1   305.352    645.431   0.473  0.64399    
## poly(r, degree = 2)2 -1781.893    456.388  -3.904  0.00181 ** 
## theta                   -2.045      4.555  -0.449  0.66091    
## theta:r                 -2.127      1.163  -1.829  0.09049 .  
## ---
## Signif. codes:  0 '***' 0.001 '**' 0.01 '*' 0.05 '.' 0.1 ' ' 1
## 
## Residual standard error: 456.4 on 13 degrees of freedom
## Multiple R-squared:  0.7265, Adjusted R-squared:  0.6424 
## F-statistic: 8.634 on 4 and 13 DF,  p-value: 0.001252
```

```
## [1] "SewnPC 0.3"
##  [1] -50000      0   2000   4000   6000   8000  10000  12000  14000  16000
## [11]  18000  20000  22000  24000  26000
## 
## Call:
## lm(formula = background_subtracted ~ poly(r, degree = 2) + theta + 
##     r:theta, data = data_mask)
## 
## Residuals:
##     Min      1Q  Median      3Q     Max 
## -843.81 -433.02  -85.02  392.41 1219.38 
## 
## Coefficients:
##                        Estimate Std. Error t value Pr(>|t|)    
## (Intercept)           6.069e+03  2.404e+02  25.243 1.98e-12 ***
## poly(r, degree = 2)1 -1.551e+03  1.020e+03  -1.521  0.15224    
## poly(r, degree = 2)2 -2.388e+03  7.213e+02  -3.311  0.00563 ** 
## theta                 4.298e-01  7.199e+00   0.060  0.95330    
## theta:r               8.402e-02  1.839e+00   0.046  0.96425    
## ---
## Signif. codes:  0 '***' 0.001 '**' 0.01 '*' 0.05 '.' 0.1 ' ' 1
## 
## Residual standard error: 721.3 on 13 degrees of freedom
## Multiple R-squared:  0.5428, Adjusted R-squared:  0.4021 
## F-statistic: 3.858 on 4 and 13 DF,  p-value: 0.02797
```

```
## [1] "Sewn 0.3"
##  [1] -50000      0   2000   4000   6000   8000  10000  12000  14000  16000
## [11]  18000  20000  22000  24000  26000
## 
## Call:
## lm(formula = background_subtracted ~ poly(r, degree = 2) + theta + 
##     r:theta, data = data_mask)
## 
## Residuals:
##      Min       1Q   Median       3Q      Max 
## -2010.21 -1051.13   -90.17  1133.26  2356.88 
## 
## Coefficients:
##                       Estimate Std. Error t value Pr(>|t|)    
## (Intercept)           4301.500    504.506   8.526 1.11e-06 ***
## poly(r, degree = 2)1  8114.951   2140.439   3.791  0.00224 ** 
## poly(r, degree = 2)2 -5427.785   1513.519  -3.586  0.00332 ** 
## theta                   55.041     15.107   3.643  0.00297 ** 
## theta:r                -10.869      3.858  -2.817  0.01454 *  
## ---
## Signif. codes:  0 '***' 0.001 '**' 0.01 '*' 0.05 '.' 0.1 ' ' 1
## 
## Residual standard error: 1514 on 13 degrees of freedom
## Multiple R-squared:  0.7168, Adjusted R-squared:  0.6296 
## F-statistic: 8.226 on 4 and 13 DF,  p-value: 0.001554
```

```
## [1] "N95L 0.3"
##  [1] -50000      0   2000   4000   6000   8000  10000  12000  14000  16000
## [11]  18000  20000  22000  24000  26000
## 
## Call:
## lm(formula = background_subtracted ~ poly(r, degree = 2) + theta + 
##     r:theta, data = data_mask)
## 
## Residuals:
##     Min      1Q  Median      3Q     Max 
## -598.29 -333.68   77.38  180.79  713.15 
## 
## Coefficients:
##                       Estimate Std. Error t value Pr(>|t|)    
## (Intercept)          7097.3056   143.4463  49.477 3.44e-16 ***
## poly(r, degree = 2)1 -633.7992   608.5911  -1.041   0.3167    
## poly(r, degree = 2)2 -947.4380   430.3389  -2.202   0.0464 *  
## theta                  -6.5520     4.2955  -1.525   0.1511    
## theta:r                -0.5877     1.0970  -0.536   0.6012    
## ---
## Signif. codes:  0 '***' 0.001 '**' 0.01 '*' 0.05 '.' 0.1 ' ' 1
## 
## Residual standard error: 430.3 on 13 degrees of freedom
## Multiple R-squared:  0.6431, Adjusted R-squared:  0.5333 
## F-statistic: 5.857 on 4 and 13 DF,  p-value: 0.006394
```

```
## [1] "N95T 0.3"
##  [1] -50000      0   2000   4000   6000   8000  10000  12000  14000  16000
## [11]  18000  20000  22000  24000  26000
## 
## Call:
## lm(formula = background_subtracted ~ poly(r, degree = 2) + theta + 
##     r:theta, data = data_mask)
## 
## Residuals:
##     Min      1Q  Median      3Q     Max 
## -245.47  -48.73   11.66   74.07  153.66 
## 
## Coefficients:
##                       Estimate Std. Error t value Pr(>|t|)    
## (Intercept)           415.7778    38.3199  10.850 6.93e-08 ***
## poly(r, degree = 2)1 -184.7561   162.5775  -1.136  0.27630    
## poly(r, degree = 2)2 -330.6695   114.9596  -2.876  0.01298 *  
## theta                   3.7657     1.1475   3.282  0.00595 ** 
## theta:r                -0.1812     0.2930  -0.618  0.54705    
## ---
## Signif. codes:  0 '***' 0.001 '**' 0.01 '*' 0.05 '.' 0.1 ' ' 1
## 
## Residual standard error: 115 on 13 degrees of freedom
## Multiple R-squared:  0.7601, Adjusted R-squared:  0.6862 
## F-statistic: 10.29 on 4 and 13 DF,  p-value: 0.0005553
```

```
## [1] "SewnF 0.3"
##  [1] -50000      0   2000   4000   6000   8000  10000  12000  14000  16000
## [11]  18000  20000  22000  24000  26000
## 
## Call:
## lm(formula = background_subtracted ~ poly(r, degree = 2) + theta + 
##     r:theta, data = data_mask)
## 
## Residuals:
##     Min      1Q  Median      3Q     Max 
## -392.11  -50.76  -39.98   81.40  525.51 
## 
## Coefficients:
##                       Estimate Std. Error t value Pr(>|t|)    
## (Intercept)          5267.4167    78.9766  66.696  < 2e-16 ***
## poly(r, degree = 2)1  449.6548   335.0695   1.342    0.203    
## poly(r, degree = 2)2  245.8332   236.9299   1.038    0.318    
## theta                 -56.6165     2.3649 -23.940 3.89e-12 ***
## theta:r                -0.5731     0.6039  -0.949    0.360    
## ---
## Signif. codes:  0 '***' 0.001 '**' 0.01 '*' 0.05 '.' 0.1 ' ' 1
## 
## Residual standard error: 236.9 on 13 degrees of freedom
## Multiple R-squared:  0.9942, Adjusted R-squared:  0.9924 
## F-statistic: 556.8 on 4 and 13 DF,  p-value: 2.172e-14
```

```
## [1] "No Mask 0.5"
##  [1] -50000      0   8000  16000  24000  32000  40000  48000  56000  64000
## [11]  72000  80000  88000  96000 104000 112000 120000
## 
## Call:
## lm(formula = background_subtracted ~ poly(r, degree = 2) + theta + 
##     r:theta, data = data_mask)
## 
## Residuals:
##    Min     1Q Median     3Q    Max 
## -19040 -11356  -7548  12532  38898 
## 
## Coefficients:
##                      Estimate Std. Error t value Pr(>|t|)    
## (Intercept)             45.83    6505.63   0.007 0.994486    
## poly(r, degree = 2)1   -31.66   27601.06  -0.001 0.999102    
## poly(r, degree = 2)2 57075.67   19516.90   2.924 0.011840 *  
## theta                 1007.95     194.81   5.174 0.000179 ***
## theta:r               -196.03      49.75  -3.940 0.001692 ** 
## ---
## Signif. codes:  0 '***' 0.001 '**' 0.01 '*' 0.05 '.' 0.1 ' ' 1
## 
## Residual standard error: 19520 on 13 degrees of freedom
## Multiple R-squared:  0.7989, Adjusted R-squared:  0.737 
## F-statistic: 12.91 on 4 and 13 DF,  p-value: 0.0001837
```

```
## [1] "Surgical 0.5"
##  [1] -50000      0   8000  16000  24000  32000  40000  48000  56000  64000
## [11]  72000  80000  88000  96000 104000 112000 120000
## 
## Call:
## lm(formula = background_subtracted ~ poly(r, degree = 2) + theta + 
##     r:theta, data = data_mask)
## 
## Residuals:
##     Min      1Q  Median      3Q     Max 
## -308.22  -56.52  -22.63   45.18  666.78 
## 
## Coefficients:
##                       Estimate Std. Error t value Pr(>|t|)   
## (Intercept)           301.2778    74.0779   4.067  0.00133 **
## poly(r, degree = 2)1 -490.7967   314.2860  -1.562  0.14238   
## poly(r, degree = 2)2   89.3397   222.2338   0.402  0.69421   
## theta                  -3.3468     2.2182  -1.509  0.15528   
## theta:r                 0.5492     0.5665   0.970  0.34998   
## ---
## Signif. codes:  0 '***' 0.001 '**' 0.01 '*' 0.05 '.' 0.1 ' ' 1
## 
## Residual standard error: 222.2 on 13 degrees of freedom
## Multiple R-squared:   0.25,  Adjusted R-squared:  0.01921 
## F-statistic: 1.083 on 4 and 13 DF,  p-value: 0.4047
```

```
## [1] "SewnPC 0.5"
##  [1] -50000      0   8000  16000  24000  32000  40000  48000  56000  64000
## [11]  72000  80000  88000  96000 104000 112000 120000
## 
## Call:
## lm(formula = background_subtracted ~ poly(r, degree = 2) + theta + 
##     r:theta, data = data_mask)
## 
## Residuals:
##     Min      1Q  Median      3Q     Max 
## -61.289 -32.554  -3.746  28.130  86.101 
## 
## Coefficients:
##                        Estimate Std. Error t value Pr(>|t|)    
## (Intercept)           198.83333   15.70348  12.662 1.09e-08 ***
## poly(r, degree = 2)1  -57.81276   66.62422  -0.868   0.4013    
## poly(r, degree = 2)2 -126.02925   47.11043  -2.675   0.0191 *  
## theta                   0.33119    0.47024   0.704   0.4937    
## theta:r                -0.07788    0.12009  -0.648   0.5280    
## ---
## Signif. codes:  0 '***' 0.001 '**' 0.01 '*' 0.05 '.' 0.1 ' ' 1
## 
## Residual standard error: 47.11 on 13 degrees of freedom
## Multiple R-squared:  0.4624, Adjusted R-squared:  0.2969 
## F-statistic: 2.795 on 4 and 13 DF,  p-value: 0.07094
```

```
## [1] "Sewn 0.5"
##  [1] -50000      0   8000  16000  24000  32000  40000  48000  56000  64000
## [11]  72000  80000  88000  96000 104000 112000 120000
## 
## Call:
## lm(formula = background_subtracted ~ poly(r, degree = 2) + theta + 
##     r:theta, data = data_mask)
## 
## Residuals:
##     Min      1Q  Median      3Q     Max 
## -858.08  -70.39  -31.46   45.65 1875.08 
## 
## Coefficients:
##                       Estimate Std. Error t value Pr(>|t|)  
## (Intercept)            474.019    207.964   2.279   0.0402 *
## poly(r, degree = 2)1 -1190.413    882.316  -1.349   0.2003  
## poly(r, degree = 2)2   329.411    623.892   0.528   0.6064  
## theta                   -7.664      6.227  -1.231   0.2402  
## theta:r                  1.483      1.590   0.933   0.3679  
## ---
## Signif. codes:  0 '***' 0.001 '**' 0.01 '*' 0.05 '.' 0.1 ' ' 1
## 
## Residual standard error: 623.9 on 13 degrees of freedom
## Multiple R-squared:  0.1768, Adjusted R-squared:  -0.07646 
## F-statistic: 0.6981 on 4 and 13 DF,  p-value: 0.6068
```

```
## [1] "N95L 0.5"
##  [1] -50000      0   8000  16000  24000  32000  40000  48000  56000  64000
## [11]  72000  80000  88000  96000 104000 112000 120000
## 
## Call:
## lm(formula = background_subtracted ~ poly(r, degree = 2) + theta + 
##     r:theta, data = data_mask)
## 
## Residuals:
##    Min     1Q Median     3Q    Max 
## -29.54 -14.81 -11.17  16.16  40.21 
## 
## Coefficients:
##                       Estimate Std. Error t value Pr(>|t|)    
## (Intercept)          235.94444    8.31259  28.384 4.42e-13 ***
## poly(r, degree = 2)1  27.45342   35.26733   0.778  0.45025    
## poly(r, degree = 2)2 -75.69702   24.93777  -3.035  0.00956 ** 
## theta                  0.02729    0.24892   0.110  0.91437    
## theta:r               -0.06930    0.06357  -1.090  0.29544    
## ---
## Signif. codes:  0 '***' 0.001 '**' 0.01 '*' 0.05 '.' 0.1 ' ' 1
## 
## Residual standard error: 24.94 on 13 degrees of freedom
## Multiple R-squared:  0.4968, Adjusted R-squared:  0.342 
## F-statistic: 3.209 on 4 and 13 DF,  p-value: 0.04871
```

```
## [1] "N95T 0.5"
##  [1] -50000      0   8000  16000  24000  32000  40000  48000  56000  64000
## [11]  72000  80000  88000  96000 104000 112000 120000
## 
## Call:
## lm(formula = background_subtracted ~ poly(r, degree = 2) + theta + 
##     r:theta, data = data_mask)
## 
## Residuals:
##      Min       1Q   Median       3Q      Max 
## -19.7164  -7.9890   0.8728   6.2025  16.7632 
## 
## Coefficients:
##                        Estimate Std. Error t value Pr(>|t|)   
## (Intercept)           16.148148   3.960248   4.078  0.00131 **
## poly(r, degree = 2)1 -43.690952  16.801907  -2.600  0.02199 * 
## poly(r, degree = 2)2 -19.823834  11.880743  -1.669  0.11909   
## theta                  0.211176   0.118588   1.781  0.09832 . 
## theta:r                0.009487   0.030285   0.313  0.75906   
## ---
## Signif. codes:  0 '***' 0.001 '**' 0.01 '*' 0.05 '.' 0.1 ' ' 1
## 
## Residual standard error: 11.88 on 13 degrees of freedom
## Multiple R-squared:  0.6936, Adjusted R-squared:  0.5993 
## F-statistic: 7.356 on 4 and 13 DF,  p-value: 0.002525
```

```
## [1] "SewnF 0.5"
##  [1] -50000      0   8000  16000  24000  32000  40000  48000  56000  64000
## [11]  72000  80000  88000  96000 104000 112000 120000
## 
## Call:
## lm(formula = background_subtracted ~ poly(r, degree = 2) + theta + 
##     r:theta, data = data_mask)
## 
## Residuals:
##     Min      1Q  Median      3Q     Max 
## -9.1667 -0.7500 -0.2083  1.2500  5.8333 
## 
## Coefficients:
##                       Estimate Std. Error t value Pr(>|t|)    
## (Intercept)          191.83333    1.56961 122.217  < 2e-16 ***
## poly(r, degree = 2)1  65.38348    6.65929   9.818 2.22e-07 ***
## poly(r, degree = 2)2   3.77492    4.70883   0.802    0.437    
## theta                 -1.85370    0.04700 -39.439 6.43e-15 ***
## theta:r               -0.08333    0.01200  -6.943 1.02e-05 ***
## ---
## Signif. codes:  0 '***' 0.001 '**' 0.01 '*' 0.05 '.' 0.1 ' ' 1
## 
## Residual standard error: 4.709 on 13 degrees of freedom
## Multiple R-squared:  0.9983, Adjusted R-squared:  0.9978 
## F-statistic:  1891 on 4 and 13 DF,  p-value: < 2.2e-16
```

```
## [1] "No Mask 1"
##  [1] -50000      0   2000   4000   6000   8000  10000  12000  14000  16000
## [11]  18000  20000  22000  24000  26000  28000  30000
## 
## Call:
## lm(formula = background_subtracted ~ poly(r, degree = 2) + theta + 
##     r:theta, data = data_mask)
## 
## Residuals:
##     Min      1Q  Median      3Q     Max 
## -5693.3 -3414.0  -975.4  3834.4  5689.6 
## 
## Coefficients:
##                       Estimate Std. Error t value Pr(>|t|)    
## (Intercept)              1.074   1628.420   0.001 0.999484    
## poly(r, degree = 2)1    -3.358   6908.799   0.000 0.999620    
## poly(r, degree = 2)2 17184.593   4885.259   3.518 0.003784 ** 
## theta                  303.486     48.763   6.224  3.1e-05 ***
## theta:r                -59.005     12.453  -4.738 0.000388 ***
## ---
## Signif. codes:  0 '***' 0.001 '**' 0.01 '*' 0.05 '.' 0.1 ' ' 1
## 
## Residual standard error: 4885 on 13 degrees of freedom
## Multiple R-squared:  0.8518, Adjusted R-squared:  0.8062 
## F-statistic: 18.68 on 4 and 13 DF,  p-value: 2.669e-05
```

```
## [1] "Surgical 1"
##  [1] -50000      0   2000   4000   6000   8000  10000  12000  14000  16000
## [11]  18000  20000  22000  24000  26000  28000  30000
## 
## Call:
## lm(formula = background_subtracted ~ poly(r, degree = 2) + theta + 
##     r:theta, data = data_mask)
## 
## Residuals:
##      Min       1Q   Median       3Q      Max 
## -1098.24  -222.67   -97.40    98.69  2634.17 
## 
## Coefficients:
##                       Estimate Std. Error t value Pr(>|t|)
## (Intercept)            415.139    289.829   1.432    0.176
## poly(r, degree = 2)1 -1995.917   1229.641  -1.623    0.129
## poly(r, degree = 2)2   749.420    869.488   0.862    0.404
## theta                  -12.913      8.679  -1.488    0.161
## theta:r                  2.511      2.216   1.133    0.278
## 
## Residual standard error: 869.5 on 13 degrees of freedom
## Multiple R-squared:  0.2517, Adjusted R-squared:  0.02151 
## F-statistic: 1.093 on 4 and 13 DF,  p-value: 0.4002
```

```
## [1] "SewnPC 1"
##  [1] -50000      0   2000   4000   6000   8000  10000  12000  14000  16000
## [11]  18000  20000  22000  24000  26000  28000  30000
## 
## Call:
## lm(formula = background_subtracted ~ poly(r, degree = 2) + theta + 
##     r:theta, data = data_mask)
## 
## Residuals:
##     Min      1Q  Median      3Q     Max 
## -2.4686 -1.5033 -0.6681  0.2794  9.9481 
## 
## Coefficients:
##                       Estimate Std. Error t value Pr(>|t|)
## (Intercept)           0.537037   1.099135   0.489    0.633
## poly(r, degree = 2)1 -2.109350   4.663233  -0.452    0.658
## poly(r, degree = 2)2 -3.178878   3.297404  -0.964    0.353
## theta                 0.004435   0.032913   0.135    0.895
## theta:r               0.001478   0.008405   0.176    0.863
## 
## Residual standard error: 3.297 on 13 degrees of freedom
## Multiple R-squared:  0.1015, Adjusted R-squared:  -0.1749 
## F-statistic: 0.3673 on 4 and 13 DF,  p-value: 0.8276
```

```
## [1] "Sewn 1"
##  [1] -50000      0   2000   4000   6000   8000  10000  12000  14000  16000
## [11]  18000  20000  22000  24000  26000  28000  30000
## 
## Call:
## lm(formula = background_subtracted ~ poly(r, degree = 2) + theta + 
##     r:theta, data = data_mask)
## 
## Residuals:
##     Min      1Q  Median      3Q     Max 
## -589.61 -109.18  -53.78   53.20 1400.80 
## 
## Coefficients:
##                       Estimate Std. Error t value Pr(>|t|)
## (Intercept)            225.565    154.123   1.464    0.167
## poly(r, degree = 2)1 -1071.397    653.890  -1.638    0.125
## poly(r, degree = 2)2   389.004    462.370   0.841    0.415
## theta                   -7.050      4.615  -1.528    0.151
## theta:r                  1.375      1.179   1.166    0.264
## 
## Residual standard error: 462.4 on 13 degrees of freedom
## Multiple R-squared:  0.2542, Adjusted R-squared:  0.02473 
## F-statistic: 1.108 on 4 and 13 DF,  p-value: 0.3941
```

```
## [1] "N95L 1"
##  [1] -50000      0   2000   4000   6000   8000  10000  12000  14000  16000
## [11]  18000  20000  22000  24000  26000  28000  30000
## 
## Call:
## lm(formula = background_subtracted ~ poly(r, degree = 2) + theta + 
##     r:theta, data = data_mask)
## 
## Residuals:
##     Min      1Q  Median      3Q     Max 
## -11.102  -3.509   1.236   2.105  13.314 
## 
## Coefficients:
##                        Estimate Std. Error t value Pr(>|t|)   
## (Intercept)            7.388889   2.219582   3.329  0.00544 **
## poly(r, degree = 2)1  -5.353034   9.416889  -0.568  0.57942   
## poly(r, degree = 2)2 -10.596259   6.658746  -1.591  0.13555   
## theta                 -0.104841   0.066465  -1.577  0.13872   
## theta:r                0.006823   0.016974   0.402  0.69425   
## ---
## Signif. codes:  0 '***' 0.001 '**' 0.01 '*' 0.05 '.' 0.1 ' ' 1
## 
## Residual standard error: 6.659 on 13 degrees of freedom
## Multiple R-squared:  0.3924, Adjusted R-squared:  0.2055 
## F-statistic: 2.099 on 4 and 13 DF,  p-value: 0.1392
```

```
## [1] "N95T 1"
##  [1] -50000      0   2000   4000   6000   8000  10000  12000  14000  16000
## [11]  18000  20000  22000  24000  26000  28000  30000
## 
## Call:
## lm(formula = background_subtracted ~ poly(r, degree = 2) + theta + 
##     r:theta, data = data_mask)
## 
## Residuals:
##    Min     1Q Median     3Q    Max 
## -2.956 -1.621 -1.026  0.932 11.044 
## 
## Coefficients:
##                       Estimate Std. Error t value Pr(>|t|)
## (Intercept)           1.777778   1.219561   1.458    0.169
## poly(r, degree = 2)1  0.152944   5.174160   0.030    0.977
## poly(r, degree = 2)2 -3.576237   3.658683  -0.977    0.346
## theta                -0.027290   0.036519  -0.747    0.468
## theta:r               0.004483   0.009326   0.481    0.639
## 
## Residual standard error: 3.659 on 13 degrees of freedom
## Multiple R-squared:  0.126,  Adjusted R-squared:  -0.1429 
## F-statistic: 0.4687 on 4 and 13 DF,  p-value: 0.7579
```

```
## [1] "SewnF 1"
##  [1] -50000      0   2000   4000   6000   8000  10000  12000  14000  16000
## [11]  18000  20000  22000  24000  26000  28000  30000
## 
## Call:
## lm(formula = background_subtracted ~ poly(r, degree = 2) + theta + 
##     r:theta, data = data_mask)
## 
## Residuals:
##     Min      1Q  Median      3Q     Max 
## -5.1751 -1.0508 -0.4722  0.9638 12.2416 
## 
## Coefficients:
##                       Estimate Std. Error t value Pr(>|t|)
## (Intercept)           2.138889   1.360903   1.572    0.140
## poly(r, degree = 2)1 -8.201612   5.773821  -1.420    0.179
## poly(r, degree = 2)2  4.232985   4.082708   1.037    0.319
## theta                -0.052956   0.040752  -1.299    0.216
## theta:r               0.009868   0.010407   0.948    0.360
## 
## Residual standard error: 4.083 on 13 degrees of freedom
## Multiple R-squared:  0.2344, Adjusted R-squared:  -0.001227 
## F-statistic: 0.9948 on 4 and 13 DF,  p-value: 0.4448
```

```
## [1] "No Mask 3"
##  [1] -50000      0    800   1600   2400   3200   4000   4800   5600   6400
## [11]   7200   8000   8800   9600  10400  11200  12000
## 
## Call:
## lm(formula = background_subtracted ~ poly(r, degree = 2) + theta + 
##     r:theta, data = data_mask)
## 
## Residuals:
##     Min      1Q  Median      3Q     Max 
## -2260.0 -1061.2  -706.5   962.8  4392.0 
## 
## Coefficients:
##                      Estimate Std. Error t value Pr(>|t|)    
## (Intercept)             1.704    657.452   0.003 0.997972    
## poly(r, degree = 2)1    1.504   2789.331   0.001 0.999578    
## poly(r, degree = 2)2 5334.907   1972.355   2.705 0.018027 *  
## theta                  94.165     19.687   4.783 0.000358 ***
## theta:r               -18.308      5.028  -3.641 0.002986 ** 
## ---
## Signif. codes:  0 '***' 0.001 '**' 0.01 '*' 0.05 '.' 0.1 ' ' 1
## 
## Residual standard error: 1972 on 13 degrees of freedom
## Multiple R-squared:  0.7724, Adjusted R-squared:  0.7024 
## F-statistic: 11.03 on 4 and 13 DF,  p-value: 0.0003992
```

```
## [1] "Surgical 3"
##  [1] -50000      0    800   1600   2400   3200   4000   4800   5600   6400
## [11]   7200   8000   8800   9600  10400  11200  12000
## 
## Call:
## lm(formula = background_subtracted ~ poly(r, degree = 2) + theta + 
##     r:theta, data = data_mask)
## 
## Residuals:
##     Min      1Q  Median      3Q     Max 
## -732.72 -145.00  -65.89   66.67 1752.61 
## 
## Coefficients:
##                       Estimate Std. Error t value Pr(>|t|)
## (Intercept)            277.741    192.827   1.440    0.173
## poly(r, degree = 2)1 -1328.445    818.095  -1.624    0.128
## poly(r, degree = 2)2   500.408    578.480   0.865    0.403
## theta                   -8.664      5.774  -1.501    0.157
## theta:r                  1.688      1.475   1.144    0.273
## 
## Residual standard error: 578.5 on 13 degrees of freedom
## Multiple R-squared:  0.2525, Adjusted R-squared:  0.02248 
## F-statistic: 1.098 on 4 and 13 DF,  p-value: 0.3984
```

```
## [1] "SewnPC 3"
##  [1] -50000      0    800   1600   2400   3200   4000   4800   5600   6400
## [11]   7200   8000   8800   9600  10400  11200  12000
## 
## Call:
## lm(formula = background_subtracted ~ poly(r, degree = 2) + theta + 
##     r:theta, data = data_mask)
## 
## Residuals:
##     Min      1Q  Median      3Q     Max 
## -2.5760 -1.6151 -0.8582  0.6758  4.8933 
## 
## Coefficients:
##                        Estimate Std. Error t value Pr(>|t|)  
## (Intercept)           2.1481481  0.8985664   2.391   0.0327 *
## poly(r, degree = 2)1  1.5804195  3.8122944   0.415   0.6852  
## poly(r, degree = 2)2 -0.4194352  2.6956992  -0.156   0.8787  
## theta                -0.0059129  0.0269073  -0.220   0.8295  
## theta:r              -0.0009422  0.0068715  -0.137   0.8930  
## ---
## Signif. codes:  0 '***' 0.001 '**' 0.01 '*' 0.05 '.' 0.1 ' ' 1
## 
## Residual standard error: 2.696 on 13 degrees of freedom
## Multiple R-squared:  0.04803,    Adjusted R-squared:  -0.2449 
## F-statistic: 0.164 on 4 and 13 DF,  p-value: 0.9529
```

```
## [1] "Sewn 3"
##  [1] -50000      0    800   1600   2400   3200   4000   4800   5600   6400
## [11]   7200   8000   8800   9600  10400  11200  12000
## 
## Call:
## lm(formula = background_subtracted ~ poly(r, degree = 2) + theta + 
##     r:theta, data = data_mask)
## 
## Residuals:
##     Min      1Q  Median      3Q     Max 
## -372.36  -73.18  -34.87   33.98  895.64 
## 
## Coefficients:
##                       Estimate Std. Error t value Pr(>|t|)
## (Intercept)           142.2593    98.5411   1.444    0.173
## poly(r, degree = 2)1 -679.7078   418.0746  -1.626    0.128
## poly(r, degree = 2)2  249.1887   295.6234   0.843    0.415
## theta                  -4.4507     2.9508  -1.508    0.155
## theta:r                 0.8712     0.7536   1.156    0.268
## 
## Residual standard error: 295.6 on 13 degrees of freedom
## Multiple R-squared:  0.2508, Adjusted R-squared:  0.02028 
## F-statistic: 1.088 on 4 and 13 DF,  p-value: 0.4026
```

```
## [1] "N95L 3"
##  [1] -50000      0    800   1600   2400   3200   4000   4800   5600   6400
## [11]   7200   8000   8800   9600  10400  11200  12000
## 
## Call:
## lm(formula = background_subtracted ~ poly(r, degree = 2) + theta + 
##     r:theta, data = data_mask)
## 
## Residuals:
##     Min      1Q  Median      3Q     Max 
## -6.4430 -1.2116 -0.0015  1.0888  6.8904 
## 
## Coefficients:
##                      Estimate Std. Error t value Pr(>|t|)    
## (Intercept)           5.74074    1.11409   5.153 0.000186 ***
## poly(r, degree = 2)1 -7.85112    4.72670  -1.661 0.120623    
## poly(r, degree = 2)2 -1.21416    3.34228  -0.363 0.722241    
## theta                -0.08980    0.03336  -2.692 0.018484 *  
## theta:r               0.01101    0.00852   1.293 0.218608    
## ---
## Signif. codes:  0 '***' 0.001 '**' 0.01 '*' 0.05 '.' 0.1 ' ' 1
## 
## Residual standard error: 3.342 on 13 degrees of freedom
## Multiple R-squared:  0.4824, Adjusted R-squared:  0.3231 
## F-statistic: 3.029 on 4 and 13 DF,  p-value: 0.05723
```

```
## [1] "N95T 3"
##  [1] -50000      0    800   1600   2400   3200   4000   4800   5600   6400
## [11]   7200   8000   8800   9600  10400  11200  12000
## 
## Call:
## lm(formula = background_subtracted ~ poly(r, degree = 2) + theta + 
##     r:theta, data = data_mask)
## 
## Residuals:
##     Min      1Q  Median      3Q     Max 
## -1.4196 -0.7233 -0.1111  0.2259  3.2471 
## 
## Coefficients:
##                        Estimate Std. Error t value Pr(>|t|)  
## (Intercept)           0.9259259  0.4016396   2.305   0.0383 *
## poly(r, degree = 2)1  0.6627566  1.7040125   0.389   0.7036  
## poly(r, degree = 2)2 -1.5673633  1.2049188  -1.301   0.2159  
## theta                -0.0080572  0.0120270  -0.670   0.5146  
## theta:r               0.0008122  0.0030714   0.264   0.7956  
## ---
## Signif. codes:  0 '***' 0.001 '**' 0.01 '*' 0.05 '.' 0.1 ' ' 1
## 
## Residual standard error: 1.205 on 13 degrees of freedom
## Multiple R-squared:  0.1947, Adjusted R-squared:  -0.05303 
## F-statistic: 0.786 on 4 and 13 DF,  p-value: 0.5544
```

```
## [1] "SewnF 3"
##  [1] -50000      0    800   1600   2400   3200   4000   4800   5600   6400
## [11]   7200   8000   8800   9600  10400  11200  12000
## 
## Call:
## lm(formula = background_subtracted ~ poly(r, degree = 2) + theta + 
##     r:theta, data = data_mask)
## 
## Residuals:
##     Min      1Q  Median      3Q     Max 
## -1.2895 -0.6733 -0.2047  0.1981  2.5292 
## 
## Coefficients:
##                       Estimate Std. Error t value Pr(>|t|)    
## (Intercept)           1.740741   0.370857   4.694  0.00042 ***
## poly(r, degree = 2)1  4.154974   1.573415   2.641  0.02037 *  
## poly(r, degree = 2)2  0.883022   1.112572   0.794  0.44163    
## theta                -0.000130   0.011105  -0.012  0.99084    
## theta:r              -0.005393   0.002836  -1.902  0.07961 .  
## ---
## Signif. codes:  0 '***' 0.001 '**' 0.01 '*' 0.05 '.' 0.1 ' ' 1
## 
## Residual standard error: 1.113 on 13 degrees of freedom
## Multiple R-squared:  0.5704, Adjusted R-squared:  0.4382 
## F-statistic: 4.315 on 4 and 13 DF,  p-value: 0.0194
```

```
## [1] "No Mask 5"
##  [1] -50000      0    600   1200   1800   2400   3000   3600   4200   4800
## [11]   5400   6000   6600   7200   7800   8400   9000   9600  10200  10800
## 
## Call:
## lm(formula = background_subtracted ~ poly(r, degree = 2) + theta + 
##     r:theta, data = data_mask)
## 
## Residuals:
##     Min      1Q  Median      3Q     Max 
## -2565.2  -907.2  -602.8   798.3  4417.8 
## 
## Coefficients:
##                      Estimate Std. Error t value Pr(>|t|)    
## (Intercept)             1.731    621.142   0.003 0.997818    
## poly(r, degree = 2)1   -1.153   2635.283   0.000 0.999657    
## poly(r, degree = 2)2 4556.038   1863.426   2.445 0.029490 *  
## theta                  80.430     18.600   4.324 0.000825 ***
## theta:r               -15.641      4.750  -3.293 0.005827 ** 
## ---
## Signif. codes:  0 '***' 0.001 '**' 0.01 '*' 0.05 '.' 0.1 ' ' 1
## 
## Residual standard error: 1863 on 13 degrees of freedom
## Multiple R-squared:  0.7351, Adjusted R-squared:  0.6536 
## F-statistic: 9.018 on 4 and 13 DF,  p-value: 0.001028
```

```
## [1] "Surgical 5"
##  [1] -50000      0    600   1200   1800   2400   3000   3600   4200   4800
## [11]   5400   6000   6600   7200   7800   8400   9000   9600  10200  10800
## 
## Call:
## lm(formula = background_subtracted ~ poly(r, degree = 2) + theta + 
##     r:theta, data = data_mask)
## 
## Residuals:
##     Min      1Q  Median      3Q     Max 
## -510.77 -103.52  -45.76   47.81 1227.32 
## 
## Coefficients:
##                      Estimate Std. Error t value Pr(>|t|)
## (Intercept)           193.824    135.031   1.435    0.175
## poly(r, degree = 2)1 -927.273    572.888  -1.619    0.130
## poly(r, degree = 2)2  346.067    405.093   0.854    0.408
## theta                  -6.032      4.043  -1.492    0.160
## theta:r                 1.172      1.033   1.135    0.277
## 
## Residual standard error: 405.1 on 13 degrees of freedom
## Multiple R-squared:  0.2509, Adjusted R-squared:  0.02045 
## F-statistic: 1.089 on 4 and 13 DF,  p-value: 0.4023
```

```
## [1] "SewnPC 5"
##  [1] -50000      0    600   1200   1800   2400   3000   3600   4200   4800
## [11]   5400   6000   6600   7200   7800   8400   9000   9600  10200  10800
## 
## Call:
## lm(formula = background_subtracted ~ poly(r, degree = 2) + theta + 
##     r:theta, data = data_mask)
## 
## Residuals:
##     Min      1Q  Median      3Q     Max 
## -2.3439 -1.1343 -0.5154  0.8214  4.8286 
## 
## Coefficients:
##                        Estimate Std. Error t value Pr(>|t|)  
## (Intercept)           1.6111111  0.7229588   2.228   0.0441 *
## poly(r, degree = 2)1 -0.2867697  3.0672544  -0.093   0.9269  
## poly(r, degree = 2)2 -2.1799595  2.1688764  -1.005   0.3332  
## theta                -0.0048246  0.0216488  -0.223   0.8271  
## theta:r              -0.0005279  0.0055286  -0.095   0.9254  
## ---
## Signif. codes:  0 '***' 0.001 '**' 0.01 '*' 0.05 '.' 0.1 ' ' 1
## 
## Residual standard error: 2.169 on 13 degrees of freedom
## Multiple R-squared:  0.09767,    Adjusted R-squared:  -0.18 
## F-statistic: 0.3518 on 4 and 13 DF,  p-value: 0.8382
```

```
## [1] "Sewn 5"
##  [1] -50000      0    600   1200   1800   2400   3000   3600   4200   4800
## [11]   5400   6000   6600   7200   7800   8400   9000   9600  10200  10800
## 
## Call:
## lm(formula = background_subtracted ~ poly(r, degree = 2) + theta + 
##     r:theta, data = data_mask)
## 
## Residuals:
##     Min      1Q  Median      3Q     Max 
## -350.11  -71.33  -32.83   32.35  835.89 
## 
## Coefficients:
##                       Estimate Std. Error t value Pr(>|t|)
## (Intercept)           132.7315    91.9660   1.443    0.173
## poly(r, degree = 2)1 -636.9027   390.1788  -1.632    0.127
## poly(r, degree = 2)2  236.5118   275.8981   0.857    0.407
## theta                  -4.1535     2.7539  -1.508    0.155
## theta:r                 0.8101     0.7033   1.152    0.270
## 
## Residual standard error: 275.9 on 13 degrees of freedom
## Multiple R-squared:  0.2533, Adjusted R-squared:  0.02358 
## F-statistic: 1.103 on 4 and 13 DF,  p-value: 0.3963
```

```
## [1] "N95L 5"
##  [1] -50000      0    600   1200   1800   2400   3000   3600   4200   4800
## [11]   5400   6000   6600   7200   7800   8400   9000   9600  10200  10800
## 
## Call:
## lm(formula = background_subtracted ~ poly(r, degree = 2) + theta + 
##     r:theta, data = data_mask)
## 
## Residuals:
##     Min      1Q  Median      3Q     Max 
## -3.9605 -1.0731  0.1754  0.9605  4.0395 
## 
## Coefficients:
##                       Estimate Std. Error t value Pr(>|t|)    
## (Intercept)           3.185185   0.733031   4.345 0.000794 ***
## poly(r, degree = 2)1 -4.027521   3.109988  -1.295 0.217838    
## poly(r, degree = 2)2 -1.103777   2.199094  -0.502 0.624114    
## theta                -0.034064   0.021950  -1.552 0.144689    
## theta:r               0.001608   0.005606   0.287 0.778720    
## ---
## Signif. codes:  0 '***' 0.001 '**' 0.01 '*' 0.05 '.' 0.1 ' ' 1
## 
## Residual standard error: 2.199 on 13 degrees of freedom
## Multiple R-squared:  0.4072, Adjusted R-squared:  0.2248 
## F-statistic: 2.232 on 4 and 13 DF,  p-value: 0.1218
```

```
## [1] "N95T 5"
##  [1] -50000      0    600   1200   1800   2400   3000   3600   4200   4800
## [11]   5400   6000   6600   7200   7800   8400   9000   9600  10200  10800
## 
## Call:
## lm(formula = background_subtracted ~ poly(r, degree = 2) + theta + 
##     r:theta, data = data_mask)
## 
## Residuals:
##     Min      1Q  Median      3Q     Max 
## -2.4357 -0.8165 -0.1535  0.5874  3.5643 
## 
## Coefficients:
##                       Estimate Std. Error t value Pr(>|t|)   
## (Intercept)           1.703704   0.556443   3.062  0.00909 **
## poly(r, degree = 2)1  7.086397   2.360789   3.002  0.01021 * 
## poly(r, degree = 2)2  1.743968   1.669330   1.045  0.31519   
## theta                 0.019818   0.016663   1.189  0.25556   
## theta:r              -0.007180   0.004255  -1.687  0.11537   
## ---
## Signif. codes:  0 '***' 0.001 '**' 0.01 '*' 0.05 '.' 0.1 ' ' 1
## 
## Residual standard error: 1.669 on 13 degrees of freedom
## Multiple R-squared:  0.4515, Adjusted R-squared:  0.2828 
## F-statistic: 2.675 on 4 and 13 DF,  p-value: 0.07934
```

```
## [1] "SewnF 5"
##  [1] -50000      0    600   1200   1800   2400   3000   3600   4200   4800
## [11]   5400   6000   6600   7200   7800   8400   9000   9600  10200  10800
## 
## Call:
## lm(formula = background_subtracted ~ poly(r, degree = 2) + theta + 
##     r:theta, data = data_mask)
## 
## Residuals:
##     Min      1Q  Median      3Q     Max 
## -2.1652 -0.8911 -0.2102  0.4203  3.8348 
## 
## Coefficients:
##                       Estimate Std. Error t value Pr(>|t|)  
## (Intercept)           1.620370   0.539146   3.005   0.0101 *
## poly(r, degree = 2)1  1.503948   2.287403   0.657   0.5223  
## poly(r, degree = 2)2  1.269344   1.617438   0.785   0.4466  
## theta                -0.011615   0.016145  -0.719   0.4846  
## theta:r              -0.001917   0.004123  -0.465   0.6497  
## ---
## Signif. codes:  0 '***' 0.001 '**' 0.01 '*' 0.05 '.' 0.1 ' ' 1
## 
## Residual standard error: 1.617 on 13 degrees of freedom
## Multiple R-squared:  0.2997, Adjusted R-squared:  0.08427 
## F-statistic: 1.391 on 4 and 13 DF,  p-value: 0.2909
```

```
## [1] "No Mask 10"
##  [1] -50000      0    200    400    600    800   1000   1200   1400   1600
## [11]   1800   2000   2200
## 
## Call:
## lm(formula = background_subtracted ~ poly(r, degree = 2) + theta + 
##     r:theta, data = data_mask)
## 
## Residuals:
##    Min     1Q Median     3Q    Max 
## -616.3 -167.2 -111.4  112.0 1078.7 
## 
## Coefficients:
##                       Estimate Std. Error t value Pr(>|t|)   
## (Intercept)            0.05556  136.13568   0.000  0.99968   
## poly(r, degree = 2)1  -0.26765  577.57477   0.000  0.99964   
## poly(r, degree = 2)2 841.11116  408.40703   2.059  0.06006 . 
## theta                 14.84717    4.07654   3.642  0.00298 **
## theta:r               -2.88582    1.04106  -2.772  0.01586 * 
## ---
## Signif. codes:  0 '***' 0.001 '**' 0.01 '*' 0.05 '.' 0.1 ' ' 1
## 
## Residual standard error: 408.4 on 13 degrees of freedom
## Multiple R-squared:  0.6631, Adjusted R-squared:  0.5594 
## F-statistic: 6.396 on 4 and 13 DF,  p-value: 0.004508
```

```
## [1] "Surgical 10"
##  [1] -50000      0    200    400    600    800   1000   1200   1400   1600
## [11]   1800   2000   2200
## 
## Call:
## lm(formula = background_subtracted ~ poly(r, degree = 2) + theta + 
##     r:theta, data = data_mask)
## 
## Residuals:
##     Min      1Q  Median      3Q     Max 
## -70.743 -14.677  -7.004   7.504 170.757 
## 
## Coefficients:
##                       Estimate Std. Error t value Pr(>|t|)
## (Intercept)            27.3333    18.7914   1.455    0.170
## poly(r, degree = 2)1 -127.5551    79.7253  -1.600    0.134
## poly(r, degree = 2)2   46.6567    56.3743   0.828    0.423
## theta                  -0.8432     0.5627  -1.498    0.158
## theta:r                 0.1674     0.1437   1.165    0.265
## 
## Residual standard error: 56.37 on 13 degrees of freedom
## Multiple R-squared:  0.2433, Adjusted R-squared:  0.01047 
## F-statistic: 1.045 on 4 and 13 DF,  p-value: 0.4216
```

```
## [1] "SewnPC 10"
##  [1] -50000      0    200    400    600    800   1000   1200   1400   1600
## [11]   1800   2000   2200
## 
## Call:
## lm(formula = background_subtracted ~ poly(r, degree = 2) + theta + 
##     r:theta, data = data_mask)
## 
## Residuals:
##      Min       1Q   Median       3Q      Max 
## -0.55044 -0.43640 -0.17982  0.05099  1.94956 
## 
## Coefficients:
##                        Estimate Std. Error t value Pr(>|t|)
## (Intercept)           2.778e-01  2.418e-01   1.149    0.271
## poly(r, degree = 2)1 -7.647e-01  1.026e+00  -0.745    0.469
## poly(r, degree = 2)2 -4.305e-01  7.255e-01  -0.593    0.563
## theta                 9.747e-05  7.242e-03   0.013    0.989
## theta:r               3.411e-04  1.849e-03   0.184    0.857
## 
## Residual standard error: 0.7255 on 13 degrees of freedom
## Multiple R-squared:  0.08757,    Adjusted R-squared:  -0.1932 
## F-statistic: 0.3119 on 4 and 13 DF,  p-value: 0.8649
```

```
## [1] "Sewn 10"
##  [1] -50000      0    200    400    600    800   1000   1200   1400   1600
## [11]   1800   2000   2200
## 
## Call:
## lm(formula = background_subtracted ~ poly(r, degree = 2) + theta + 
##     r:theta, data = data_mask)
## 
## Residuals:
##     Min      1Q  Median      3Q     Max 
## -80.996 -15.840  -7.947   8.281 191.504 
## 
## Coefficients:
##                       Estimate Std. Error t value Pr(>|t|)
## (Intercept)            31.5000    21.0724   1.495    0.159
## poly(r, degree = 2)1 -144.0731    89.4028  -1.612    0.131
## poly(r, degree = 2)2   55.0343    63.2173   0.871    0.400
## theta                  -0.9559     0.6310  -1.515    0.154
## theta:r                 0.1838     0.1611   1.141    0.275
## 
## Residual standard error: 63.22 on 13 degrees of freedom
## Multiple R-squared:  0.2541, Adjusted R-squared:  0.02462 
## F-statistic: 1.107 on 4 and 13 DF,  p-value: 0.3943
```

```
## [1] "N95L 10"
##  [1] -50000      0    200    400    600    800   1000   1200   1400   1600
## [11]   1800   2000   2200
## 
## Call:
## lm(formula = background_subtracted ~ poly(r, degree = 2) + theta + 
##     r:theta, data = data_mask)
## 
## Residuals:
##     Min      1Q  Median      3Q     Max 
## -1.5833 -0.9375 -0.2500  0.7500  2.5000 
## 
## Coefficients:
##                       Estimate Std. Error t value Pr(>|t|)   
## (Intercept)           1.611111   0.456825   3.527  0.00372 **
## poly(r, degree = 2)1 -0.191180   1.938146  -0.099  0.92293   
## poly(r, degree = 2)2 -0.397360   1.370476  -0.290  0.77643   
## theta                -0.016667   0.013680  -1.218  0.24473   
## theta:r               0.001852   0.003493   0.530  0.60498   
## ---
## Signif. codes:  0 '***' 0.001 '**' 0.01 '*' 0.05 '.' 0.1 ' ' 1
## 
## Residual standard error: 1.37 on 13 degrees of freedom
## Multiple R-squared:  0.1696, Adjusted R-squared:  -0.08593 
## F-statistic: 0.6637 on 4 and 13 DF,  p-value: 0.6282
```

```
## [1] "N95T 10"
##  [1] -50000      0    200    400    600    800   1000   1200   1400   1600
## [11]   1800   2000   2200
## 
## Call:
## lm(formula = background_subtracted ~ poly(r, degree = 2) + theta + 
##     r:theta, data = data_mask)
## 
## Residuals:
##      Min       1Q   Median       3Q      Max 
## -1.39474 -0.15351 -0.09211  0.08443  2.15351 
## 
## Coefficients:
##                        Estimate Std. Error t value Pr(>|t|)  
## (Intercept)           0.7777778  0.3049671   2.550   0.0242 *
## poly(r, degree = 2)1  2.2176854  1.2938659   1.714   0.1103  
## poly(r, degree = 2)2 -0.4635863  0.9149014  -0.507   0.6208  
## theta                 0.0007797  0.0091322   0.085   0.9333  
## theta:r              -0.0028265  0.0023321  -1.212   0.2471  
## ---
## Signif. codes:  0 '***' 0.001 '**' 0.01 '*' 0.05 '.' 0.1 ' ' 1
## 
## Residual standard error: 0.9149 on 13 degrees of freedom
## Multiple R-squared:  0.3315, Adjusted R-squared:  0.1258 
## F-statistic: 1.612 on 4 and 13 DF,  p-value: 0.2302
```

```
## [1] "SewnF 10"
##  [1] -50000      0    200    400    600    800   1000   1200   1400   1600
## [11]   1800   2000   2200
## 
## Call:
## lm(formula = background_subtracted ~ poly(r, degree = 2) + theta + 
##     r:theta, data = data_mask)
## 
## Residuals:
##     Min      1Q  Median      3Q     Max 
## -1.3903 -1.0161 -0.1857  0.8637  3.1096 
## 
## Coefficients:
##                       Estimate Std. Error t value Pr(>|t|)  
## (Intercept)           1.222222   0.498406   2.452   0.0291 *
## poly(r, degree = 2)1 -0.152944   2.114559  -0.072   0.9434  
## poly(r, degree = 2)2  0.640191   1.495219   0.428   0.6755  
## theta                -0.017154   0.014925  -1.149   0.2711  
## theta:r               0.002924   0.003811   0.767   0.4567  
## ---
## Signif. codes:  0 '***' 0.001 '**' 0.01 '*' 0.05 '.' 0.1 ' ' 1
## 
## Residual standard error: 1.495 on 13 degrees of freedom
## Multiple R-squared:  0.1396, Adjusted R-squared:  -0.1252 
## F-statistic: 0.5271 on 4 and 13 DF,  p-value: 0.7179
```
